# Unveiling novel antioxidant peptides from silver carp (*Hypophthalmichthys molitrix*) bone collagen and underlying multi-dimensional mechanism by integrating *in silico*, *in vitro*, and *in cellulo* approaches

**DOI:** 10.1016/j.fochx.2026.104070

**Published:** 2026-06-06

**Authors:** Yimeng Mei, Feng Lin, Ruoyu Xie, Jiaxin Chen, Jun Hu, Wenxuan Chen, Hongying Du, Guijie Hao, Shuangxi Li, Jin Zhang

**Affiliations:** a State Key Laboratory for Quality and Safety of Agro-products, Zhejiang Key Laboratory of Intelligent Food Logistic and Processing, Institute of Food Science, Zhejiang Academy of Agricultural Sciences, Hangzhou, Zhejiang 310021, PR China; b Department of Food Science and Engineering, College of Light Industry and Food Engineering, Nanjing Forestry University, Nanjing 210037, PR China; c Key Laboratory of Healthy Freshwater Aquaculture, Ministry of Agriculture and Rural Affairs, Key Laboratory of Fish Health and Nutrition of Zhejiang Province, Huzhou Key Laboratory of Aquatic Product Quality Improvement and Processing Technology, Zhejiang Institute of Freshwater Fisheries, Huzhou, Zhejiang 313001, PR China; d Xingzhi College, Zhejiang Normal University, Lanxi, Zhejiang 321100, PR China

**Keywords:** Antioxidant peptide, Silver carp, Bone, Collagen, *In silico* exploration, *In vitro* assessment, *In cellulo* validation

## Abstract

This work has achieved, for the first time, the unveiling of novel antioxidant peptides from silver carp bone collagen (SCBC) and their underlying multi-dimensional mechanism by integrating *in silico*, *in vitro*, and *in cellulo* approaches. AEDVN, EDDR, and DVEL were identified from SCBC with good DPPH**·**-scavenging activity (IC_50_ = 0.89–2.59 mM), strong Fe^2+^-chelating ability (IC_50_ = 0.98–3.72 mM), and high stability against simulated gastrointestinal digestion. The acylamino, guanidinium, and N-terminal amino groups can be their dominant redox active sites, respectively. They could form strong hydrogen bonds and electrostatic attractions with core residues (such as ARG380, ARG415, and ARG483) of Kelch domain within Keap1, thereby activating the Keap1-Nfr2-ARE antioxidant pathway. Therefore, the activities of superoxide dismutase, catalase, and glutathione peroxidase were significantly increased, resulting in the malondialdehyde reduction and cell viability improvement against H_2_O_2_-induced oxidative damage. These findings provided a cost-effective and time-saving strategy for high-value utilization of fish by-product bones as natural antioxidant sources.

## Highlights

• Antioxidant peptides from silver carp bone collagen and their mechanism were unveiled.
• Three novel antioxidant peptides (AEDVN, EDDR, and DVEL) were screened and identified.
• Peptides up-regulated SOD, CAT, and GSH-Px in H_2_O_2_-induced oxidatively damaged cells.
• Acylamino, guanidinium, and N1 amino groups were their key redox sites, respectively.
• AEDVN showed highest DPPH-scavenging and Fe^2+^-chelating abilities (IC_50_ = 894–983 μM).

## 1 Introduction

Antioxidants play a crucial role in protecting the human body against oxidative stress and thus preventing various chronic diseases, such as diabetes, inflammation, hypertension, neurodegeneration, and atherosclerosis (Wang, Danzeng, et al., 2025; Zhang et al., 2020). Mechanistically, antioxidants could perform their activity both directly and indirectly (Mubango et al., 2025). Directly, they can scavenge free radicals and chelate transition metal ions (Li et al., 2025). Indirectly, some antioxidants could relieve oxidative stress by activating the Kelch-like ECH-associated protein 1 (Keap1)-nuclear factor erythroid 2-related factor 2 (Nrf2)-antioxidant response element (ARE) pathway (Zhang, Li, et al., 2021; Zou et al., 2023). Specifically, these antioxidants competitively bind to Keap1 and block Keap1-Nrf2 interaction, which suppresses ubiquitination and degradation of Nrf2 (Halliwell, 2024). This allows Nrf2 to translocate into the nucleus, bind to ARE, and thereby trigger antioxidant-related gene transcription, thereby enhancing cellular resistance to oxidation (Mubango et al., 2025). Currently, many antioxidants have been chemically synthesized for commercial uses, such as propyl gallate, butylated hydroxyanisole, and tert-butyl hydroquinone, but most of them are usually accompanied by side effects unfavorable for long-term use (Halliwell, 2024). Hence, as a low-molecular-weight antioxidant derived from natural sources, the antioxidative peptide became one of their safer alternatives. Recently, it has been gaining remarkably increasing attractions due to its multi-target regulatory capacities, non-toxicity, minimal adverse effects, and favorable organoleptic quality compared with synthetic antioxidants (Igbokwe et al., 2024; Mubango et al., 2025).

Silver carp (*Hypophthalmichthys molitrix*) is a bulk freshwater fish species indigenous to Asian-Pacific countries such as Indonesia, America, and China, and its annual aquaculture production has exceeded 3.86 million tons in China (Zu et al., 2025). Silver carp is mostly used to produce surimi in industry due to its large production, low value, and unsatisfactory taste, but large amounts of by-products are generated during this process, mainly including bones, scales, skins, and maws (Mubango et al., 2025; Zu et al., 2025). Silver carp bones, accounting for around 13% of fish fresh weight and rich in various nutrients such as collagen and calcium, are also regarded as potential low-cost, sustainable, and natural sources of proteins and bioactive peptides (Mubango et al., 2025; Zhang et al., 2017). However, only parts of them are used as raw materials in fish meal production, most of them are still wasted or discarded, leading to potential environmental pollution and economic value reduction of the freshwater fish industry (Zhang et al., 2017). Additionally, collagen has been considered a good source of antioxidant peptides due to its unique sequence composition and relatively strong hydrophobicity (Mardani et al., 2023). As collagens account for >80% of total proteins in fish bones (Zhang et al., 2017), collagen-derived antioxidant peptides have been explored and identified from diverse fish bone sources, such as skipjack tuna bone (Ding et al., 2019), tilapia bone (Luo et al., 2022), and sturgeon bone (Zhang, Gui, et al., 2024). Nevertheless, bioactive peptides from silver carp bone collagen (SCBC) were rarely investigated, and the potential of SCBC to produce antioxidant peptides has not been reported yet.

On the other hand, the traditional technical route to develop bioactive peptides based on *in vivo* approaches is usually time-consuming, high-cost, and unkind to animals (Zhang, Wu, et al., 2024). Recently, large numbers of *in silico* rapid approaches, mainly including the simulated hydrolysis/digestion, AI-aided virtual screening, molecular simulation, quantum chemical computation, and network pharmacology, are increasingly utilized in the unveiling of food-derived novel bioactive peptides and mechanistic understanding from multi-dimensional perspectives such as the molecule, cell, and human body levels (Igbokwe et al., 2024; Liu, Guo, et al., 2022; Wang et al., 2024; Zhao et al., 2021). Meanwhile, Huang et al. (2024) and Gao et al. (2025) reported that the *in silico* explored hemp seed protein-derived xanthine oxidase inhibitory peptides and whey protein-derived bi-functional peptides were highly consistent in sequences with those directly explored, respectively. Moreover, a combined strategy of *in vitro* and *in cellulo* approaches has also been developed as a cost-effective, risk-reduced, and animal-welfare alternative to *in vivo* approaches for the biosecurity and bioactivity evaluation (Zhang, Li, et al., 2021; Zhang, Wu, et al., 2024). Furthermore, Li et al. (2024) investigated the antioxidant activity and gastrointestinal stability of soy peptide-based nanoparticles through *in silico*, *in vitro*, and *in cellulo* evaluations, respectively, and the results also showed high consistence among different approaches. Therefore, this study aimed to unveil novel antioxidant peptides from SCBC and their underlying multi-dimensional mechanism by integrating *in silico*, *in vitro*, and *in cellulo* approaches. The information obtained from this work could provide a cost-effective and time-saving strategy for the high-value development of fish by-products as natural sources of antioxidants.

## 2 Materials and methods

### 2.1 Materials

1,1-diphenyl-2-picrylhydrazyl (DPPH), trifluoroacetic acid (TFA), ferrozine, and bovine serum albumin (BSA) were purchased from China National Medicines Co., Ltd. (Beijing, China). The salivary α-amylase (100 U/mg), pepsin (3000 U/g), α-chymotrypsin (≥45 U/mg), and trypsin (2500 U/mg) were purchased from Yuanye Biotechnology Co., Ltd. (Shanghai, China). The Bicinchoninic Acid (BCA) Protein assay kit, Dulbecco's modified eagle medium (DMEM), fetal bovine serum (FBS), and penicillin-streptomycin were purchased from Thermo Fisher Scientific (Madison, WI, USA). The assay kits of methyl thiazolyl tetrazolium (MTT), superoxide dismutase (SOD), catalase (CAT), glutathione peroxidase (GSH-Px), and malondialdehyde (MDA) were bought from Solarbio Life Sciences (Beijing, China). The HepG2 cells were obtained from Beijing Institute of Biochemistry and Cell Biology (Beijing, China). All chemicals and reagents used were of analytical reagent grade.

### 2.2 *In silico* simulated proteolysis of SCBC and peptide release analysis

The α1(I)-chain and α2(I)-chain sequences of SCBC were obtained from the UniProt Knowledgebase (http://www.uniprot.org/) with the accession numbers of A0A077B3P8 and A0A2H4ZEX8, respectively. Afterward, the SignalP 5.0 online server (https://services.healthtech.dtu.dk/services/SignalP-5.0/) was employed to predict the signal peptides of selected protein sequences based on deep neural networks (Almagro Armenteros et al., 2019), and signal sequences with the highest probabilities were removed. According to the method of Zhang, Wu, et al. (2024) with some modifications, the simulated proteolysis of SCBC with selected sequences was performed using the “Enzyme(s) Action” tool of BIOPEP-UWM database (https://biochemia.uwm.edu.pl/biopep-uwm/). Firstly, the selected SCBC sequences were subjected to the virtual hydrolysis of single enzyme using 27 representative proteases, including chymotrypsin A (EC: 3.4.21.1), trypsin (EC: 3.4.21.4), pepsin (EC: 3.4.23.1, pH = 1.3), proteinase K (EC: 3.4.21.67), pancreatic elastase (EC: 3.4.21.36), prolyl oligopeptidase (EC: 3.4.21.26), thermolysin (EC: 3.4.24.27), chymotrypsin C (EC: 3.4.21.2), plasmin (EC: 3.4.21.7), cathepsin G (EC: 3.4.21.20), clostripain (EC: 3.4.22.8), chymase (EC: 3.4.21.39), papain (EC: 3.4.22.2), ficin (EC: 3.4.22.3), leukocyte elastase (EC: 3.4.21.37), metridin (EC: 3.4.21.3), thrombin (EC: 3.4.21.5), pancreatic elastase II (EC: 3.4.21.71), stem bromelain (EC: 3.4.22.32), oligopeptidase B (EC: 3.4.21.83), calpain 2 (EC: 3.4.22.53), glycyl endopeptidase (EC: 3.4.22.25), oligopeptidase F, proteinase P1 (EC: 3.4.21.96), Xaa-Pro dipeptidase (EC: 3.4.13.9), coccolysin (EC: 3.4.24.30), and glutamyl endopeptidase (EC: 3.4.21.19, pH = 7.8). The frequency of fragments with antioxidative capacity (*A*_*antioxidative*_) and theoretical degree of hydrolysis (*DH*) were calculated with the following equations (Zhang, Wu, et al., 2024):

(1) $$A_{\mathrm{antioxidative}}=\frac{a_{\mathrm{antioxidative}}}{N}$$

(2) $$\mathrm{DH}=\frac{d}{D}\times 100\%$$

where *A*_*antioxidative*_ represented the frequency of fragments with antioxidative activity in a sequence, *a*_*antioxidative*_ was the number of fragments with antioxidative activity of this sequence, and *N* was the number of amino acid residues of this sequence; meanwhile, *DH* represented the theoretical degree of hydrolysis for protein sequence, *d* was the number of proteolyzed peptide bonds in the sequence, and *D* was the total number of peptide bonds of the sequence.

Then the proteases with relatively high *A*_*antioxidative*_ for both α1(I)-chain and α2(I)-chain sequences were chosen for a combined enzymolysis (two- or three-proteases combinations) to obtain the maximum amount of probable antioxidant peptide fragments from SCBC. Afterward, the sequences of obtained peptides were compared with known antioxidative peptides from the BIOPEP-UWM database and previously peer-reviewed reports. Furthermore, the novel peptide sequences (unreported with antioxidation by either BIOPEP-UWM database or previously peer-reviewed studies) were submitted to the ToxinPred (http://crdd.osdd.net/raghava/toxinpred/) and AllergenFP V1.0 (https://ddg-pharmfac.net/AllergenFP/) web tools to predict the potential toxicity and allergenicity, respectively, based on the method of Liu, Guo, et al. (2022). For the sake of food safety, only novel peptides predicted as both non-toxin and non-allergen were selected for the further investigation.

### 2.3 Molecular docking between selected peptides and Keap1

The molecular docking of selected peptides with Keap1 was performed using the Discovery Studio V2019 (DS2019) software (Dassault Systèmes Biovia, San Diego, CA, USA) following the procedures of Zhang, Li, et al. (2021) with minor modifications. Specifically, the crystal structure of human Keap1 (PDB ID: 2FUL) was obtained from RCSB Protein Data Bank (PDB) (https://www1.rcsb.org/). This 3D structure was pretreated with the “Prepare Protein” program of DS2019 for water molecules removal, cocrystallized ligands (mainly Nrf2 16-mer peptide) removal, loops building, hydrogen atoms addition, and energy minimization. The docking pocket was defined based on the coordinates of Nrf2 16-mer peptide as x: 5, y: 9, and z: 1 with a radius of 15 Å (Zhang, Li, et al., 2021). Furthermore, the (6aS,7S,10aS)-8-hydroxy-4methoxy-2,7,10a-trimethyl-5,6,6a,7,10,10a-hexahydrobenzo[*h*]quinazoline-9‑carbonitrile (TX6, PubChem Compound ID: 121488089) was employed as a reference ligand because of its strong capacity to bind with Keap1 and activate the Keap1-Nrf2-ARE pathway (Huerta et al., 2016). Meanwhile, the structures of screened peptides were constructed through the “Build and Edit Protein” tool in DS2019. Then the 3D peptide structures were pretreated with the “Smart Minimizer” algorithm in DS2019 to add hydrogen atoms, input CHARMm forcefield, and minimize the energy.

Afterward, the semi-flexible CDOCKER protocol in DS2019 was conducted for the molecular docking analysis. Briefly, the receptor (human Keap1) was set as rigid and the ligands (screened peptides and TX6) were set as flexible. Results were evaluated based on the –CDOCKER energy (–CE) and –CDOCKER interaction energy (–CIE) of optimal docking conformation for each ligand-receptor interaction. The 3D and 2D diagrams with binding sites and non-bonding interactional modes were also obtained to help understand the potential antioxidative mechanism of peptides through the Keap1-Nrf2-ARE pathway.

### 2.4 Virtual screening of novel antioxidant peptide candidates

According to the methods of Zhang et al. (2020) and Wu et al. (2023) with some modifications, the selected peptides were virtually screened based on three key factors, including the binding affinity with Keap1, free radical scavenging capacity, and transition metal ion chelating activity. Specifically, the underlying binding affinity with Keap1 can be expressed as the –CIE value through molecular docking as mentioned above, while the free radical-scavenging (FRS) potential and transition metal ion-chelating (CHEL) potential were calculated using the AnOxPePred V1.0 online tool (https://services.healthtech.dtu.dk/services/AnOxPePred-1.0/) following a deep learning procedure (Olsen et al., 2020). The total scores of selected peptides were determined with the following equation:

(3) Total Score=ScoreA×50%+ScoreB×25%+ScoreC×25%

where *Score A* was the min-max linear normalized value of –CIE for molecular docking with Keap1 and mapped to 60–100; *Score B* was the min-max linear normalized value of FRS and mapped to 80–100; and *Score C* was the min-max linear normalized value of CHEL and also mapped to 80–100. *Score A* represented the indirect antioxidant activity and was thereby endowed with 50% weight of the total score, while *Score B* and *Score C* were associated with the direct antioxidant capacity, equally sharing the other 50% weight. Moreover, peptides with a total score ≥ 90 were regarded as the novel antioxidant peptide candidates.

### 2.5 Solid-phase synthesis of novel antioxidant peptide candidates

According to the method of Zhang, Wu, et al. (2024), the screened novel antioxidant peptide candidates were synthesized through a Fmoc-protected amino acid synthesis method following a solid-phase procedure. The purity of synthesized peptide was detected using a high-performance liquid chromatograph (HPLC) (Agilent 1260, Agilent Technologies Inc., Santa Clara, CA, USA), while the molecular mass of synthesized peptide was determined by the mass spectrometry (MS) (5600 plus, AB SCIEX, Foster City, CA, USA). Only synthesized peptides with the purity validated >95% (*w*/w) can be used for the further analysis.

### 2.6 *In vitro* assessment of antioxidant activity

#### 2.6.1 DPPH radical-scavenging capacity

The DPPH radical-scavenging activity was measured following the method of Li et al. (2025) and Zhang, Li, et al. (2021) with slight modifications. Briefly, a 0.1 mM DPPH ethanolic solution (*w*/*v*) was prepared with the anhydrous ethanol solvent and kept in the dark before the study. The sample group comprised of 1 mL sample solution with 4 mL DPPH solution, the composition of control group was 1 mL sample solution and 4 mL anhydrous ethanol, while the blank group contained 1 mL deionized water mixed with 4 mL DPPH solution. Subsequently, the absorbance of the reaction mixture was determined at 517 nm using a microplate reader (SpectraMax 190, Molecular Devices, San Jose, CA, USA) after blending and keeping in the dark for 30 min at room temperature. The DPPH radical-scavenging activity of synthesized peptide was calculated based on the following equation:

(4) $$\mathrm{DPPH radical scavening capacity}\;\left(\%\right)=\left[1-\left(A_{s}-A_{c}\right)/A_{b}\right]\times 100\%$$

where the *A*_*b*_, *A*_*c*_, and *A*_*s*_ represented the absorbance of the blank group, control group, and sample group, respectively.

#### 2.6.2 Fe^2+^-chelating ability

The Fe^2+^-chelating ability was determined as described by Li et al. (2025) and Zhang et al. (2020) with minor modifications. Specifically, the sample group comprised of 1 mL sample solution mixed with 3.7 mL deionized water, 0.1 mL 2 mM FeCl_2_ solution, and 0.2 mL 5 mM ferrozine solution; the control group contained 1 mL sample solution and 4 mL deionized water; and the composition of blank group was 4.7 mL deionized water mixed with 0.1 mL 2 mM FeCl_2_ solution and 0.2 mL 5 mM ferrozine solution. After vortexed and kept at room temperature for 20 min, the absorbance of mixture was detected at 562 nm. The Fe^2+^-chelating ability of synthesized peptide was calculated using the equation as follows:

(5) $${\mathrm{Fe}}^{2+}\;\mathrm{chelating ability}\;\left(\%\right)=\left[1-\left(A_{s}-A_{c}\right)/A_{b}\right]\times 100\%$$

where the *A*_*b*_, *A*_*c*_, and *A*_*s*_ represented the absorbance of the blank group, control group, and sample group, respectively.

#### 2.6.3 Calculation of half-maximal inhibitory concentration

As described by Zhang et al. (2020), the half-maximal inhibitory concentration (IC_50_) was used to compare the antioxidant activities among different synthesized peptides with glutathione (GSH) as the control. It was obtained *via* the non-linear regression analysis by fitting with the sigmoidal dose-response equation as follows:

(6) y=100/10^lgIC50−lgx×Hill slope+1

where *y* was the DPPH radical scavenging or Fe^2+^ chelating rate of test peptide, %; *x* was the concentration of test peptide, mg/mL or mM; IC_50_ represented the half-maximal inhibitory concentration of testing peptide, mg/mL or mM.

### 2.7 In cellulo validation of toxicity and antioxidant activity

According to the methods of Zhang, Li, et al. (2021) and Zou et al. (2023) with some modifications, the toxicity and antioxidant activity of peptides were further *in cellulo* validated using a H_2_O_2_-induced oxidatively damaged HepG2 cell model. For the cytotoxic detection of identified peptides, HepG2 cells were seeded in a 6-well plate with DMEM containing 10% FBS and 1% penicillin-streptomycin, and incubated at 37 °C under 5% CO_2_ for 24 h (SCO6WE, Sheldon Manufacturing Inc., Cornelius, OR, USA). Afterward, the cells were incubated with peptide solutions at different concentrations for a further 24 h, and then the cell survival was determined at 570 nm using the microplate reader following the MTT assay protocol. For the cytoprotective detection against oxidative damage, the procedure differences with cytotoxic detection focused on the incubation mixtures and detection indexes. Specifically, the incubation mixture of control group only included 100 μL cell suspension, that of H_2_O_2_ groups involved 100 μL cell suspension +100 μL H_2_O_2_ solution, whereas those of peptide-protected groups with three peptides × three levels involved 100 μL cell suspension +50 μL H_2_O_2_ solution +50 μL peptide solution. After the final incubation, cells from each group were collected, homogenized in phosphate buffer solution for 60 s in an ice bath, and then centrifuged at 10,000*g* for 10 min at 4 °C. Afterward, the protein concentration in the supernatant was measured using the BCA Protein assay kit with BSA as a standard. The levels of CAT, MDA, SOD, and GSH-Px were then determined with assay kits following the manufacturer's instructions. Besides, the final concentrations of HepG2 cells, H_2_O_2_, and peptides were 1.0 × 10^4^ cells/well, 500 μM, and 200–1000 μM, respectively, for all groups of both cytotoxic and cytoprotective detections if added.

### 2.8 Stability against simulated gastrointestinal digestion

Based on the procedures of Wang, Xu, et al. (2025) and Zhang et al. (2023) with some modifications, the stabilities of identified novel antioxidant peptides against simulated gastrointestinal digestion (SGID) were evaluated using a three-stage *in vitro* digestion model. For the simulated salivary digestion (SSD), the peptide was added to 5 mL solution containing 100 U/mL α-amylase (pH = 6.8), followed by an incubation at 37 °C and 100 rpm for 3 min (COS-100B, Bilon Instruments Co., Ltd., China). For the simulated gastric digestion (SGD), the peptide digesta was further adjusted to pH 2.0 and mixed with 5 mL solution containing 2000 U/mL pepsin (pH = 2.0), followed by an incubation at 37 °C and 100 rpm for 2 h. While for the simulated intestinal digestion (SID), after further adjusted to pH 7.5, the peptide digesta was added with 10 mL solution containing 100 U/mL trypsin and 25 U/mL α-chymotrypsin (pH = 7.5), followed by an incubation at 37 °C and 100 rpm for 2 h. After SSD, SGD, or SID, the peptide digesta was incubated at 95 °C for 20 min to inactivate the enzyme, followed by a centrifugation at 4 °C and 9000 rpm. Then the supernatant was collected for the assessment of DPPH radical-scavenging and Fe^2+^-chelating activities as described in Section 2.6*.*

### 2.9 Characterization of physicochemical and pharmacokinetic properties

The physicochemical properties of identified novel antioxidant peptides and other candidates were obtained as described by Zhang et al. (2020) and Zhang, Wu, et al. (2024) with slight modifications. The source, location, and molecular weight (MW) of peptides were acquired from the results of Sections 2.2 and 2.5. The hydrophobicity, net charge, and isoelectric point (pI) of peptides were computed with the PepDraw server (http://pepdraw.com/). In addition, the sensory quality was analyzed *via* the “Sensory Peptides and Amino Acids” server and “Calculations” tool of BIOPEP-UWM database. Besides, the water solubility was predicted by the iDrug online platform (https://drug.ai.tencent.com/en/) based on a thermodynamic solubility calculation.

Moreover, as described by Zhao et al. (2021) and Zhang, Wu, et al. (2024) with some modifications, the pharmacokinetic properties of identified peptides and other candidates were *in silico* characterized using the iDrug, which mainly included the following absorption, distribution, metabolism, and excretion (ADME) parameters: i) absorption: human intestinal absorption (HIA) probability, human oral bioavailability (HOB), P-glycoprotein (P-gp) substrate probability, and P-gp inhibition probability; ii) distribution: plasma protein binding (PPB) and blood-brain barrier permeation (BBBP) probability; iii) metabolism: cytochrome P450 (CYP450) substrate probability, CYP450 isoforms (CYP1A2, CYP2C19, CYP2C9, CYP2D6, and CYP3A4) inhibition probability, human liver microsomal metabolism (HLM) stability; and iv) excretion: human clearance.

### 2.10 Quantum chemical calculation and active site distribution

The quantum chemical calculation of identified peptides and other candidates was performed with the GaussView V5.0 and Gaussian 16 W programs (Gaussian Inc., Wallingford, CT, USA) following the procedures of Igbokwe et al. (2024) and Mubango et al. (2025) with minor modifications. Briefly, the density functional theory (DFT) was employed based on a B3LYp/6–31 G (d, p) basis set for the energy computational calculation of highest occupied molecular orbital (HOMO) and lowest unoccupied molecular orbital (LUMO), which could geometrically account for the structure-activity relationship and active site distribution of peptides. Furthermore, the global quantum descriptors of peptides, including ionization energy (*I*), electron affinity (*A*), chemical potential (*μ*), electronegativity (*χ*), chemical hardness (*η*), chemical softness (*S*), and electrophilicity (*ω*), were obtained according to the following formulas:

(7) $$I=-E_{\mathrm{HOMO}}$$

(8) $$A=-E_{\mathrm{LUMO}}$$

(9) $$\mu =\left(E_{\mathrm{HOMO}}+E_{\mathrm{LUMO}}\right)/2$$

(10) $$\chi =\left(-E_{\mathrm{HOMO}}-E_{\mathrm{LUMO}}\right)/2$$

(11) $$\eta =\left(E_{\mathrm{LUMO}}-E_{\mathrm{HOMO}}\right)/2$$

(12) $$S=1/\left(E_{\mathrm{LUMO}}-E_{\mathrm{HOMO}}\right)$$

(13) $$\omega ={\left(E_{\mathrm{HOMO}}+E_{\mathrm{LUMO}}\right)}^{2}/4\left(E_{\mathrm{LUMO}}-E_{\mathrm{HOMO}}\right)$$

where *E*_*HOMO*_ and *E*_*LUMO*_ were the HOMO and LUMO energies, respectively; *I* was the ionization energy; *A* was the electron affinity; *μ* was the chemical potential; *η* and *S* were the chemical hardness and softness, respectively; *χ* and *ω* were the electronegativity and electrophilicity, respectively.

### 2.11 Potential antioxidative targets and PPI network analysis

The potential antioxidative targets in the human body of identified peptides were evaluated by the SwissTargetPrediction webtool (http://www.swisstargetprediction.ch/) and Genecards database (https://www.genecards.org/) based on the procedures of Zhao et al. (2021) and Zhang et al. (2020) with some modifications. Briefly, the SMILES files of identified peptides were imported into the SwissTargetPrediction webtool with the organism set as “*Homo sapiens*”. Then the data of antioxidation-associated targets were collected from the Genecards database with the keyword of “antioxidant” and relevance score > 1.0. The potential antioxidative targets were obtained by mapping the potential targets of identified peptides to the antioxidation-associated targets using the Venn website (https://bioinfogp.cnb.csic.es/tools/venny/).

According to the method of Zhao et al. (2021) with minor modifications, the protein-protein interaction (PPI) network of identified peptides was then constructed through the Search Tool for Retrieval of Interacting Genes (STRING) online database (http://string-db.org/) based on the overlapped targets among identified peptides and antioxidation from the Venn diagram. Specifically, the species and minimum required interaction score were set to “*Homo sapiens*” and “highest confidence” (>0.9), respectively. Then the results were imported into the Cytoscape software for the network visualization and analysis, allowing for the topological properties calculation and PPI network diagram generation. The CytoHubba plugin was used to predict the top 10 significant targets based on 8 topological analysis algorithms (MCC, MNC, EPC, Degree, Closeness, Betweenness, Radiality, and Stress). The hub targets were identified as the intersected targets from the above 8 different algorithms.

### 2.12 GO and KEGG pathway enrichment analysis

The analyses of Gene ontology (GO) and Kyoto encyclopedia of genes and genomes (KEGG) pathway enrichments were conducted using the DAVID database (https://davidbioinformatics.nih.gov/home.jsp/) following the procedure of Zhao et al. (2021) with minor modifications. The potential antioxidative targets of identified peptides were inputted with the identifier, organism, and *P* value set as “official gene symbol”, “*Homo Sapiens*”, and < 0.01, respectively. The visualization of GO enriched terms (biological processes (BP), cellular components (CC), and molecular functions (MF), Top 10 based on target counts for each category) and KEGG enriched pathways (Top 20 based on enrichment factors) were performed through the BIOINFORMATICS online platform (https://www.bioinformatics.com.cn/).

### 2.13 Statistical analysis

All experiments were performed with three biological samples and the results were shown as means ± standard deviation. The technical analysis was also carried out in triplicate for each biological sample. Figures were drawn by the Origin V2021 software (Origin-Lab, Northampton, NC, USA) and Microsoft PowerPoint 2016, whereas Tables were made with the Microsoft Excel 2016. The analyses of variance (ANOVA) and non-linear regression were performed using the SAS V9.2 software (SAS Institute Inc., Carry, NC, USA), while the hierarchical clustering analysis (HCA) was conducted by the plug-in application in Origin V2021. Differences among mean values were established with the Duncan multiple range test. The statistically significant difference was confirmed when *p* < 0.05.

## 3 Results and discussion

### 3.1 Simulated proteolysis of SCBC

**Fig. 1:**
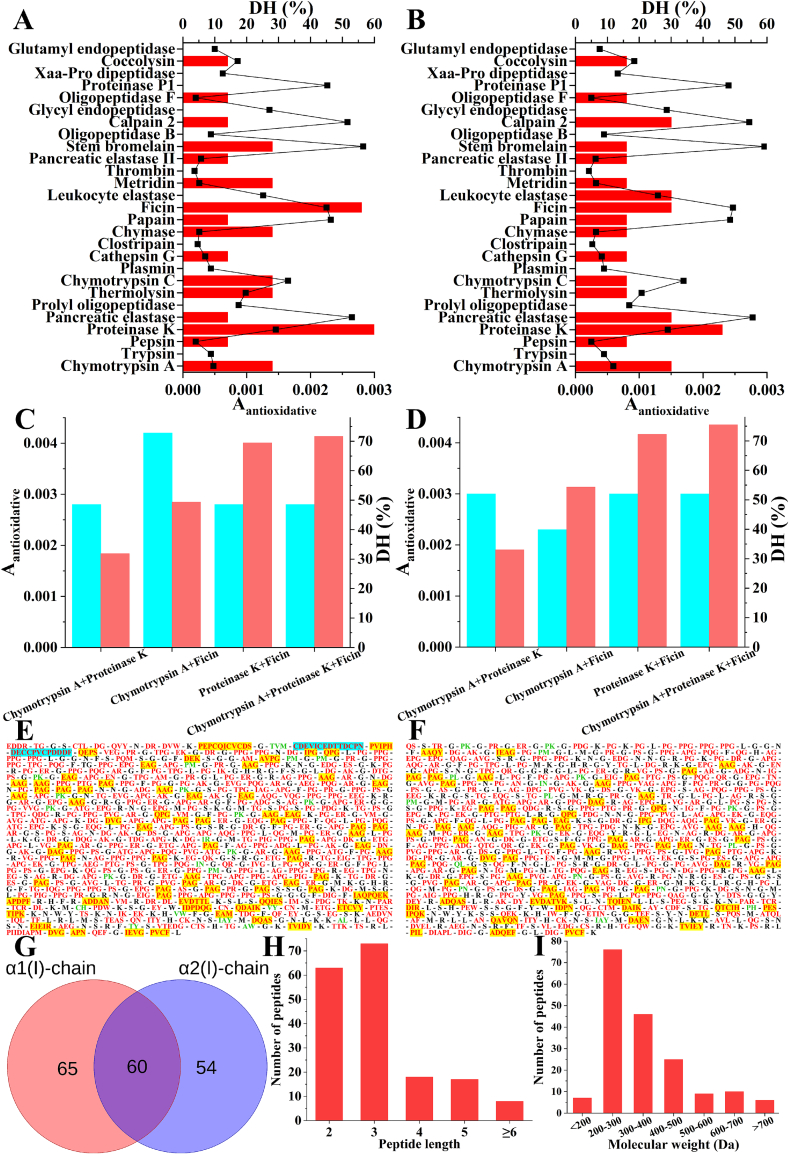
Frequency of antioxidant peptides (*A*_*antioxidative*_), degree of hydrolysis (*DH*), and peptide release analysis of SCBC after simulated proteolysis. (A–B) *A*_*antioxidative*_ and *DH* of α1(I)-chain (A) and α2(I)-chain (B) from SCBC subjected to enzymolysis with single protease, respectively. (C–D) *A*_*antioxidative*_ and *DH* of α1(I)-chain (C) and α2(I)-chain (D) from SCBC subjected to enzymolysis with combined proteases, respectively. The *A*_*antioxidative*_ and *DH* values were presented as columns and scatters, respectively. (E–F) Detailed peptide release profile from α1(I)-chain and α2(I)-chain of SCBC, respectively, after combined enzymolysis by chymotrypsin A and ficin. The known antioxidant peptides included by the BIOPEP-UWM database or reported by previously peer-reviewed studies were shown in a green color, whereas peptides with unknown antioxidant activity were indicated in a red color. The potential toxic peptides were marked with a yellow background, while the probable allergenic were labelled with an indigo background. (G–I) Sources (G), length distribution (H), and molecular weight (MW) distribution (I) of released peptides, respectively. (For interpretation of the references to color in this figure legend, the reader is referred to the web version of this article.)

Type-I collagen is known as the primary type of collagen in animal bones, accounting for around 90–95% (*w*/w) of total bone collagens (Zhang et al., 2017). Meanwhile, type-I collagen is a triple-helical structure constituted by two α1(I) chains and one α2(I) chain (Liu, Guo, et al., 2022). In the present study, the α1(I)-chain and α2(I)-chain sequences of SCBC were obtained with signal peptide sequences cut-off, and 27 representative proteases from industrial use with different known cleavage sites were utilized for their simulated proteolysis. To evaluate the proteolytic effects, *A*_*antioxidative*_ and theoretical *DH* of sequences were calculated, respectively, and the results are illustrated in Fig. 1A-B. 11 among the selected 27 proteases, including trypsin, prolyl oligopeptidase, plasmin, clostripain, leukocyte elastase, thrombin, oligopeptidase B, glycyl endopeptidase, proteinase P1, Xaa-Pro dipeptidase, and glutamyl endopeptidase, probably couldn't allow α1(I)-chain and/or α2(I)-chain to release antioxidant fragments. Meanwhile, the *A*_*antioxidative*_ values were not obviously dependent on the *DH* values for both α1(I)-chain and α2(I)-chain under the enzymolysis of different single proteases. Accordantly, Islam et al. (2021) also found that the antioxidative capacity of grass turtle muscle enzymolysates didn't exhibit a significant correlation with their DH under different proteolytic conditions.

Considering type-I collagen consists of two α1(I)-chains and one α2(I)-chain (Liu, Guo, et al., 2022), the average *A*_*antioxidative*_ values for each chain of type-I collagen were then calculated and ranked. Three proteases exhibited the best single-protease hydrolytic effects according to the average *A*_*antioxidative*_ values, which followed the order of proteinase K (average *A*_*antioxidative*_ = 0.0031, average *DH* = 29.07%) > ficin (average *A*_*antioxidative*_ = 0.0024, average *DH* = 46.49%) > chymotrypsin A (average *A*_*antioxidative*_ = 0.0014, average *DH* = 10.33%). Consistently, Panjaitan et al. (2022) reported that the proteinase K hydrolysates of mackerel (*Scomber australasicus*) steaming juice showed significantly higher antioxidative activities, including both Fe^2+^ chelating rate and reducing powder, in comparison to those hydrolyzed by papain, pepsin, or bromelain. Afterward, a combined enzymolysis with these three proteases (two- or three-protease combinations) was conducted to further promote the release of potential antioxidant fragments, and the results are exhibited in Fig. 1C-D. Generally, the combined-proteases hydrolysis increased the *DH* values of both α1(I)-chain and α2(I)-chain compared with the single-protease hydrolysis, but the *A*_*antioxidative*_ values may not further improve with the increase of *DH*, especially for the protease combinations containing proteinase K. It is probably attributed to the over-hydrolysis under combined proteolysis, leading to the cleavage of potential bioactive fragments into free amino acids and inactive shorter peptides (Wu et al., 2023). Moreover, among all two- and three-proteases combinations, “chymotrypsin A + ficin” contributed to the highest average *A*_*antioxidative*_ for each chain, which reached 0.0036 with a *DH* of 51.00%. This finding revealed that a combined proteolysis by chymotrypsin A and ficin at their appropriate conditions could be the optimum enzymolytic method to prepare antioxidant peptides from SCBC.

### 3.2 Peptide release and virtual screening

The peptide release profiles for α1(I)-chain and α2(I)-chain of SCBC under the simulated proteolysis of “chymotrypsin A + ficin” are displayed in Fig. 1E and Fig. 1F, respectively. Meanwhile, the sources, length distribution, and MW distribution of released peptides are shown in Fig. 1G-I. There were 125 and 114 peptides released from the α1(I)-chain and α2(I)-chain, respectively, among which 60 peptides were found in both hydrolysates of the two chains from SCBC (Fig. 1G). Moreover, most of the released peptides (around 70%) were dipeptides and tripeptides with a MW of 200–400 Da, followed by tetrapeptides and pentapeptides with a MW of 400–600 Da (about 20%) (Fig. 1H-I). Furthermore, five dipeptides and one tripeptide among the released peptides, including IR, TY, VY, AW, VW, and IAY, were recorded with antioxidative activity in the BIOPEP-UWM database (Fig. 1E-F). Besides, seven peptides were reported with antioxidant abilities by previously peer-reviewed studies, which included IN (IC_50_ = 1.30 mg/mL against DPPH radicals), PM (IC_50_ not shown), CH (IC_50_ < 14 μM against ABTS radicals), AL (IC_50_ not shown), TVM (IC_50_ = 0.54 mg/mL against DPPH radicals), and PK (IC_50_ = 0.5–1.0 mg/mL against superoxide radicals), and PH (IC_50_ = 0.5–1.0 mg/mL against superoxide radicals) (Liang et al., 2020; Estévez et al., 2022; Zhang, Noisa, & Yongsawatdigul, 2021; Guo et al., 2009; Wang et al., 2024). Meanwhile, among the released peptides with unknown antioxidation, 46 peptides were predicted with a certain possibility of allergenicity (Fig. 1E-F). In addition, two relatively long peptides from the α1(I)-chain, CDEVICEDTTDCPN and DECCPVCPDDDF, were predicted with high probability of toxicity (Fig. 1E).

Hence, after removing the known/reported antioxidant peptides, possibly allergenic peptides, and probably toxic peptides, a total of 119 peptides (P1-P119) derived from SCBC were selected for virtual screening, and their sequences are presented in Table S1. The virtual screening of selected peptides was conducted based on their potential to bind with Keap1, scavenge free radicals, and chelate transition metal ions, which are the three most important acting paths of antioxidants, including both direct and indirect routes (Mubango et al., 2025; Zou et al., 2023). These peptides were also scored and ranked according to their comprehensive potentials to perform antioxidation, with results also shown in Table S1. Overall, seven peptides among all selected peptides eventually exhibited the highest total scores (>90), involving P1, P10, P37, P53, P54, P94, and P98, which were considered as the SCBC-derived novel antioxidant peptide candidates.

### 3.3 Synthesis and *in vitro* evaluation of antioxidant activity

**Fig. 2:**
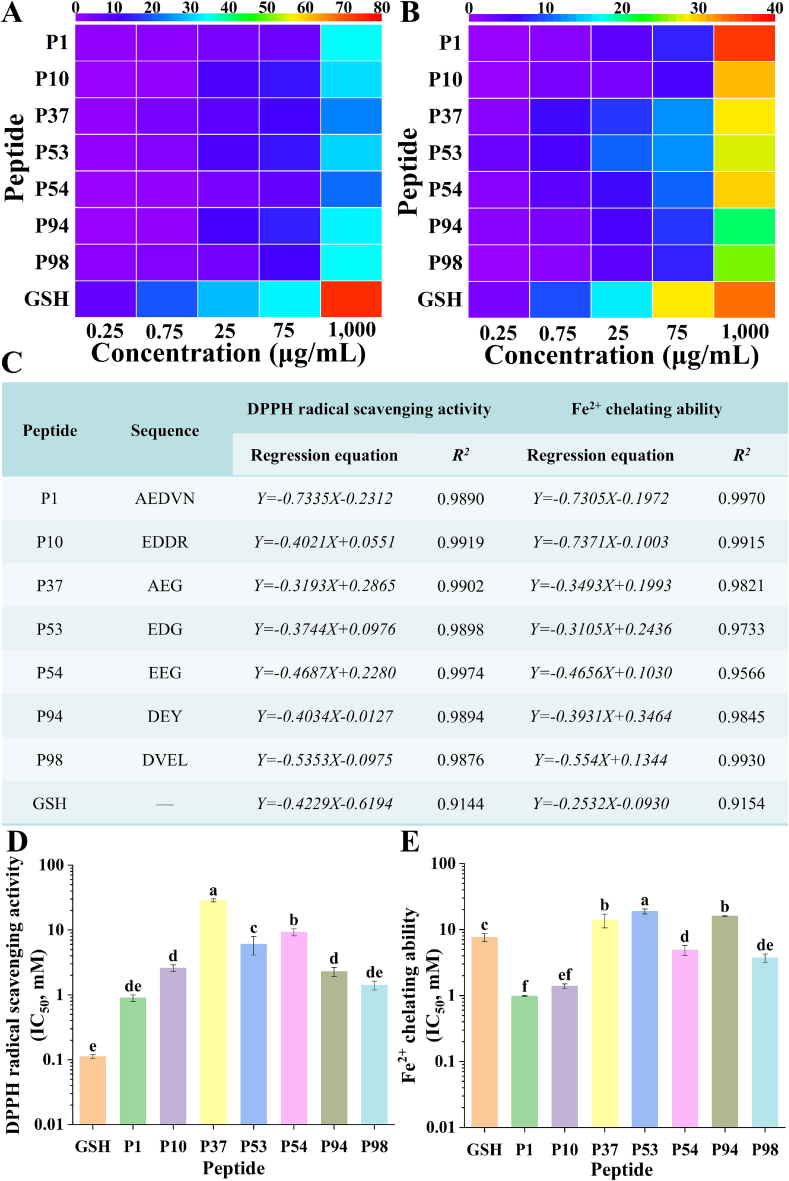
*In vitro* evaluation of antioxidant activity for synthesized novel antioxidant peptide candidates derived from SCBC. (A–B) DPPH radical-scavenging (A) and Fe^2+^-chelating (B) rates of synthesized peptides and GSH at different concentrations (0.25–1000 μg/mL), respectively. (C) Parameters fitted to the data of peptide dose-antioxidant effect relationship. *Y* = lg (100/*y* − 1) and *X* = lg *x*. *x* represented the concentration (mg/mL) of test peptides, whereas *y* represented the DPPH radical-scavenging rate (%) or Fe^2+^-chelating rate (%). (D–E) Half-maximal inhibitory concentrations (IC_50_, mM) of synthesized peptides and GSH scavenging DPPH radicals (D) and chelating Fe^2+^ (E), respectively. Different lowercases above the error bar indicated significant differences among various groups (*p* < 0.05).

These seven peptide candidates were then synthesized following a Fmoc-protected solid-phase synthetic procedure. The HPLC chromatograms and MS spectra of synthesized peptides are shown in Fig. S1. The purities of all synthesized peptides were determined within 95.50–98.17% by the HPLC-MS system. Then the antioxidations of synthesized peptides at different concentrations were *in vitro* chemically evaluated through DPPH radical-scavenging and Fe^2+^-chelating measurements, with results displayed in Fig. 2A and Fig. 2B, respectively. These data were then fitted with the sigmoidal dose-response formula (Eq. 6) by non-linear regression analysis, and the regression equations along with fitting parameters were exhibited in Fig. 2C. The *R*^*2*^ values of regression equations for all synthesized peptides were remarkably over 0.95, implying that both of their DPPH radical-scavenging ability and Fe^2+^-chelation capacity at different concentrations well conformed to the dose-response relationship. Their IC_50_ values against DPPH radicals and Fe^2+^ were further calculated from the regression equations, with results presented in Fig. 2D and Fig. 2E, respectively.

As illustrated in Fig. 2D, the control GSH was the strongest scavenger against DPPH radicals among all test peptides (IC_50_ = 0.11 ± 0.01 mM) (*p* < 0.05), followed by P1 (IC_50_ = 0.89 ± 0.10 mM) and P98 (IC_50_ = 1.40 ± 0.22 mM) (*p* < 0.05). Although weaker than that of GSH, the DPPH radical-scavenging activities of P1 and P98 were still stronger than many previously identified antioxidant peptides, such as IN (IC_50_ = 5.30 mM), IREADIDGDGQVN (IC_50_ = 1.78 mM), and ELWKTF (IC_50_ = 1.84 mM) (Liang et al., 2020; Zhang et al., 2019; Zhang et al., 2020). In addition, Tao et al. (2018) identified peptide AEVG with DPPH radical-scavenging activity (IC_50_ = 2.30 mg/mL) from cartilage protein hydrolysate of spotless smoothhound, which has a similar sequence to P1. Meanwhile, P10 and P94 exhibited the second-strongest DPPH radical-scavenging level with an IC_50_ of 2.59 ± 0.31 mM and 2.29 ± 0.36 mM, respectively (*p* < 0.05). Besides, P53 and P54 showed relatively high IC_50_ values of 6.01 ± 1.91 and 9.26 ± 1.16 mM, respectively (*p* < 0.05), indicating that they were moderate or weak scavengers against DPPH radicals. However, P37 exhibited the highest IC_50_ of 28.64 ± 1.49 mM (*p* < 0.05), suggesting the practical inactivity as a potential DPPH radical-scavenger.

Furthermore, as shown in Fig. 2E, P1 and P10 revealed the highest Fe^2+^ chelation among all test peptides (IC_50_ = 0.98 ± 0.02 and 1.39 ± 0.11 mM, respectively) (*p* < 0.05), even higher than that of some reported antioxidant peptides, such as SDGSNIHFPN (IC_50_ = 4.60 mM) and SNLCRPCG (IC_50_ = 2.18 mM) (Wan et al., 2016; Zhang, Li, et al., 2021). P98 and P54 possessed the secondly and thirdly highest Fe^2+^-chelating levels with IC_50_ values of 3.72 ± 0.53 and 4.88 ± 0.85 mM, respectively (*p* < 0.05), followed by the control GSH (IC_50_ = 7.65 ± 1.02 mM) (*p* < 0.05). Nevertheless, the other three peptides, including P37, P53, and P94, had an IC_50_ > 10 mM (*p* < 0.05), suggesting that they were practically inactive Fe^2+^ chelators. Overall, compared with the control GSH, P1, P10, and P98 displayed both good DPPH radical-scavenging activity and strong Fe^2+^-chelating capacity (Fig. 2). Hence, these three peptides were preliminarily identified as SCBC-derived novel antioxidant peptides.

### 3.4 In cellulo validation of toxicity and antioxidant activity

**Fig. 3:**
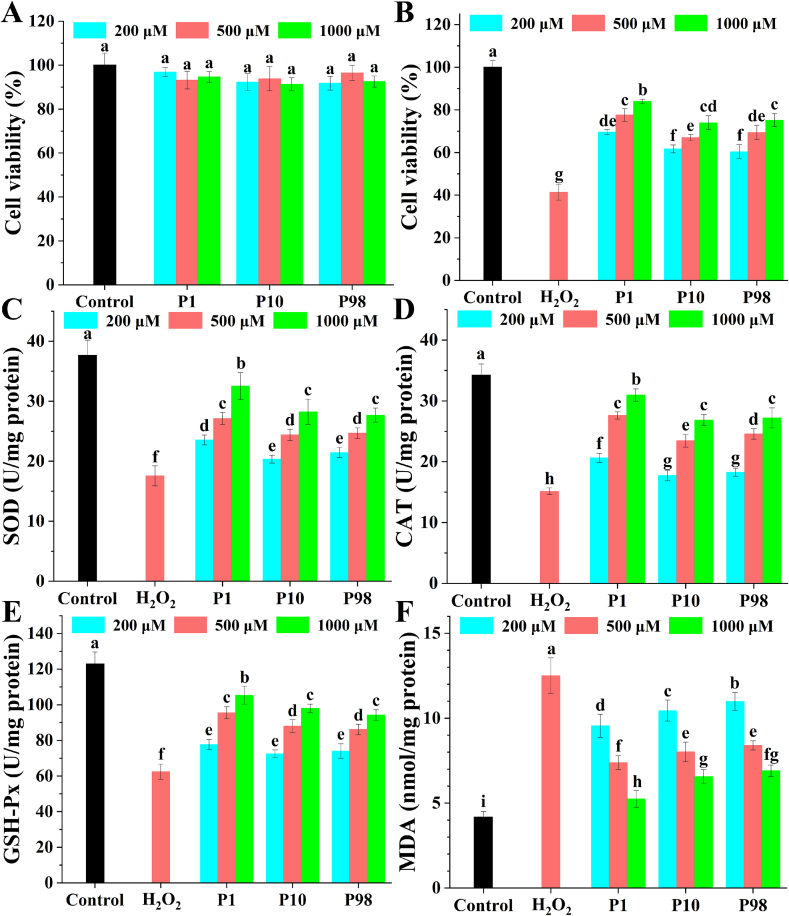
*In cellulo* validation of antioxidant activity for identified novel antioxidant peptides derived from SCBC. (A) Effects of synthetic identified peptides on cell viability of HepG2 cells. (B–F) Effects of synthetic identified peptides on cell viability (B), superoxide dismutase (SOD) activity (C), catalase (CAT) activity (D), glutathione peroxidase (GSH-Px) activity (E), and malondialdehyde (MDA) content (F) of H_2_O_2_-induced oxidatively damaged HepG2 cells. Different lowercases above the error bar indicated significant differences among various groups (*p* < 0.05).

To further validate the toxicity and antioxidant activity of P1, P10, and P98, the normal and H_2_O_2_-induced oxidatively damaged HepG2 cell models were used for the assessment, and results are presented in Fig. 3. Fig. 3A shows the effects of synthesized P1, P10, and P98 at different concentrations on cell viability of normal HepG2 cells. There were not significant differences among the cell viabilities of all groups (*p* > 0.05), demonstrating that all these identified antioxidant peptides were non-cytotoxic at the applied concentrations (200–1000 μM), which was consistent with the toxicity prediction results during peptides virtual screening (Fig. 1E-F). Besides, Fig. 3B displays the effects of identified peptides on the cell viability of H_2_O_2_-treated HepG2 cells. H_2_O_2_ penetrated cell membranes and caused cellular oxidative damage (Zhang, Li, et al., 2021), leading to a dramatic decrease in cell survival to 41.40 ± 3.70% (*p* < 0.05). However, the identified antioxidant peptides allowed the cell survival to increase prominently to 60.39–84.01% (*p* < 0.05), validating their antioxidant activity through protecting cells from H_2_O_2_-induced oxidative stress.

In addition, three key downstream enzymes of the Keap1-Nrf2-ARE antioxidant pathway, including SOD, CAT, and GSH-Px, can be activated by oxidative stress conditions (Zou et al., 2023). Meanwhile, the production of MDA, which resulted from the attack of reactive oxygen species (ROS), is also a sign of cellular oxidative stress (Zou et al., 2023). The effects of synthetic identified peptides on levels of SOD, CAT, GSH-Px, and MDA of H_2_O_2_-induced oxidatively damaged HepG2 cells are illustrated in Fig. 3C-F, respectively. These peptides obviously decreased the cellular MDA content (*p* < 0.05), whereas they markedly increased the activities of SOD, CAT, and GSH-Px (*p* < 0.05), indirectly suggesting that the identified antioxidant peptides could activate the Nrf2-ARE pathway to produce antioxidant enzymes, thereby reducing the cellular damage caused by oxidative stress. Therefore, these results further validated the non-cytotoxicity and antioxidation of P1, P10, and P98. Moreover, their overall cytoprotective effects against oxidative damage followed the order of P1 > P10 = P98 (Fig. 3B-F) (*p* < 0.05), similar with their DPPH radical-scavenging activities and Fe^2+^-chelating abilities (Fig. 2), which suggested a stronger comprehensive antioxidant activity of P1 than those of P10 and P98.

### 3.5 Stability of identified peptides against SGID

**Fig. 4:**
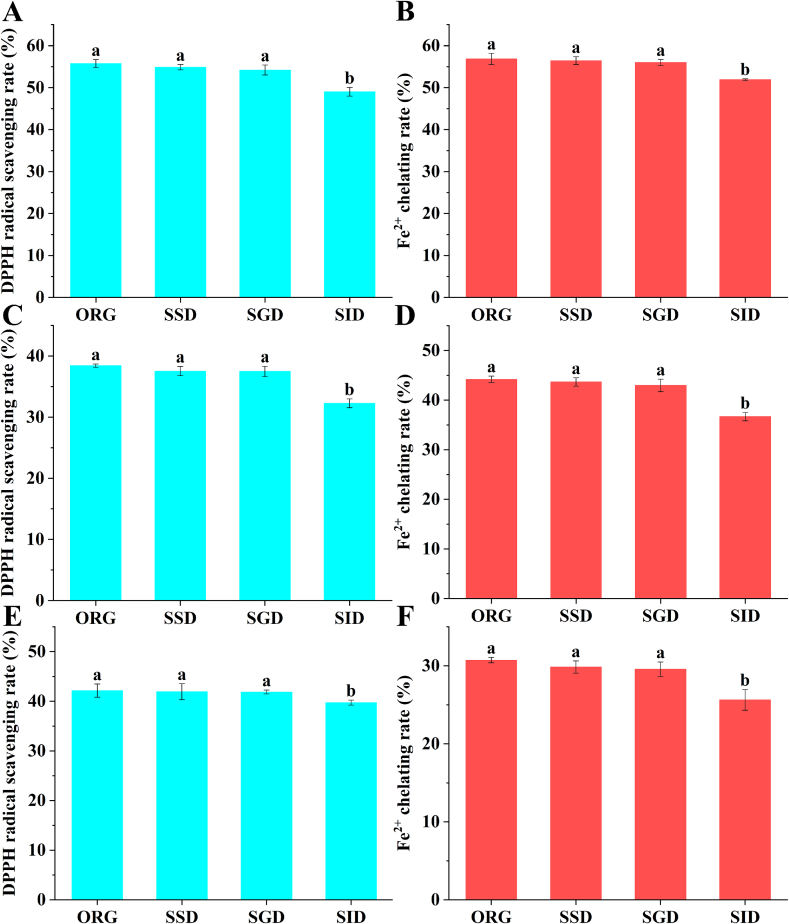
Stability of identified SCBC-derived novel antioxidant peptides against SGID. (A, C, and E) DPPH radical-scavenging rate changes of P1, P10, and P98 during SGID, respectively. (B, D, and F) Fe^2+^-chelating rate changes of P1, P10, and P98 during SGID, respectively. The concentration of all peptides used in the detection was adjusted to 1 mM, and different lowercases above the error bar indicated significant differences among various digestion phases (*p* < 0.05). ORG, original peptide; SSD, simulated salivary digestion; SGD, simulated gastric digestion; SID, simulated intestinal digestion.

The activity changes of identified novel antioxidant peptides during SGID are illustrated in Fig. 4. Both DPPH radical-scavenging and Fe^2+^-chelating capacities of P1, P10, and P98 didn't show obvious changes during SSD and SGD (*p* > 0.05), revealing particular good activity stability of these peptides against salivary and gastric digestions. Moreover, after the SID phase, their DPPH radical-scavenging and Fe^2+^-chelating abilities showed a decline of only 2.41–6.71% and 4.97–7.52%, respectively, compared with those of original samples (*p* < 0.05). Similarly, Wang, Xu, et al. (2025) also reported a decrease of only 3.2% for milk tofu cheese-derived novel antioxidant peptide NQFLPYPYY in DPPH radical-scavenging capacity during SID. In summary, these results manifested an 82.99–94.29% remaining of *in vitro* antioxidant activities for P1, P10, and P98 after the whole SGID (*p* < 0.05), suggesting their overall high resistance to gastrointestinal environments, which can be associated with their relatively short length (4–5 residues) and conducive to their application as functional food ingredients.

### 3.6 Physicochemical and pharmacokinetic properties of identified peptides

The physicochemical properties of identified novel antioxidant peptides are presented in Table S2 and compared with those of other candidates. The sequence length (4–5 residues) and MW (450–550 Da) of P1, P10, and P98 were obviously larger than those of other candidates (3 residues, 250–450 Da). Correspondingly, previous studies indicated that typical antioxidant peptides usually contain 2–20 amino acid residues and have a MW of 250–1800 Da (Mardani et al., 2023). However, the other physicochemical properties of identified peptides were similar to those of other candidates. All identified peptides showed a relatively high hydrophobicity (>10 kcal/mol), which is regarded as a typical characteristic of antioxidant peptides, allowing them to readily enter tissues and then scavenge free radicals (Mubango et al., 2025; Zhang, Li, et al., 2021). Besides, they exhibited a pI far below 7.0 (2.9–3.7) and 2 negative net charges in the neutral solvent, providing the ability to bind with Keap1 by electrostatic interactions (Huerta et al., 2016; Zhang, Li, et al., 2021). Meanwhile, they revealed a common taste of umami, which could be attributed to their high content of umami residues D and E (40–75%). Similarly, Zhang, Zhou, et al. (2024) found that the umami peptides identified from Huangjiu lees protein hydrolysates commonly revealed antioxidation against DPPH radicals. Furthermore, these peptides were predicted with a water solubility of 21.38–109.65 mM, indicating an acceptable water-soluble potential to perform antioxidation in food application, since most antioxidant peptides with good property have an IC_50_ < 10 mM (Liu, Guo, et al., 2022).

The pharmacokinetic properties of identified novel antioxidant peptides are shown in Table 1, which were *in silico* characterized and also compared with those of other candidates. Most pharmacokinetic ADME properties of P1, P10, and P98 were similar to those of other candidates, excluding HOB and PPB. Specifically, the identified peptides had a lower HOB and higher PPB in comparison to the other candidates, implying that targeted delivery strategies such as encapsulation might be required to improve their bioavailability (Zhang, Wu, et al., 2024). Meanwhile, the identified peptides and other candidates mostly had a HIA probability of >0.80, suggesting a generally good absorption in the human body. Besides, the P-gp plays a key role in cellular metabolism by transferring substrates from intracellular to extracellular (Mubango et al., 2025). All identified peptides displayed a relatively low P-gp substrate (≤0.25) but high P-gp inhibition (>0.50) probability, indicating that they were practically not substrates but inhibitors of P-gp, which were beneficial to their intracellular retention (Wu et al., 2023). Additionally, CYP450 is one of the most important enzyme families associated with pharmic metabolism in the human body (Zhao et al., 2021). The probabilities of CYP450 substrate and inhibition for identified peptides were all ≤0.25, demonstrating that they were not substrates or inhibitors of CYP450 isoforms, which revealed safety and minimal risk to human metabolism (Mubango et al., 2025). Overall, the identified peptides possessed relatively low MW, high hydrophobicity, several net charges, good water solubility, pleasant sensory taste, and favorable pharmacokinetic properties, which were all beneficial for them to perform as antioxidants.

**Table 1:** Pharmacokinetic ADME properties of identified SCBC-derived novel antioxidant peptides compared with other candidates.

Property		P1	P10	P37	P53	P54	P94	P98
Sequence		AEDVN	EDDR	AEG	EDG	EEG	DEY	DVEL
Absorption	HIA probability	0.84	0.58	1.00	1.00	1.00	0.99	0.99
	HOB (%)	13	9	39	27	24	17	14
	P-gp substrate probability	0.25	0.17	0.04	0.04	0.04	0.33	0.22
	P-gp inhibition probability	0.90	0.54	0.63	0.52	0.52	0.70	0.92
Distribution	PPB (%)	49	39	23	28	29	46	49
	BBBP probability	0.25	0.14	0.75	0.64	0.62	0.20	0.19
Metabolism	CYP450 substrate probability	0.20	0.16	0.16	0.17	0.17	0.23	0.18
	CYP1A2 inhibition probability	0.14	0.20	0.07	0.02	0.03	0.21	0.21
	CYP2C19 inhibition probability	0.12	0.25	0.13	0.07	0.08	0.16	0.13
	CYP2C9 inhibition probability	0.05	0.10	0.04	0.01	0.01	0.08	0.08
	CYP2D6 inhibition probability	0.06	0.14	0.04	0.01	0.02	0.07	0.07
	CYP3A4 inhibition probability	0.12	0.11	0.05	0.02	0.03	0.09	0.10
	HLM stability (μL/min/mg)	2.72	0.55	1.02	0.65	0.48	0.40	1.55
Excretion	Human clearance (mL/min/kg)	8.71	4.57	5.25	4.07	5.01	5.37	4.47

HIA, human intestinal absorption; HOB, human oral bioavailability; P-gp, PPB, plasma protein binding; BBBP, blood-brain barrier permeation; P-glycoprotein; HLM, human liver microsomal metabolism; CYP450, cytochrome P450; CYP1A2, CYP2C19, CYP2C9, CYP2D6, and CYP3A4, isoforms of cytochrome P450; SCBC, silver carp bone collagen; ADME, absorption, distribution, metabolism, and excretion.

### 3.7 Frontier molecular orbitals analysis of identified peptides

**Fig. 5:**
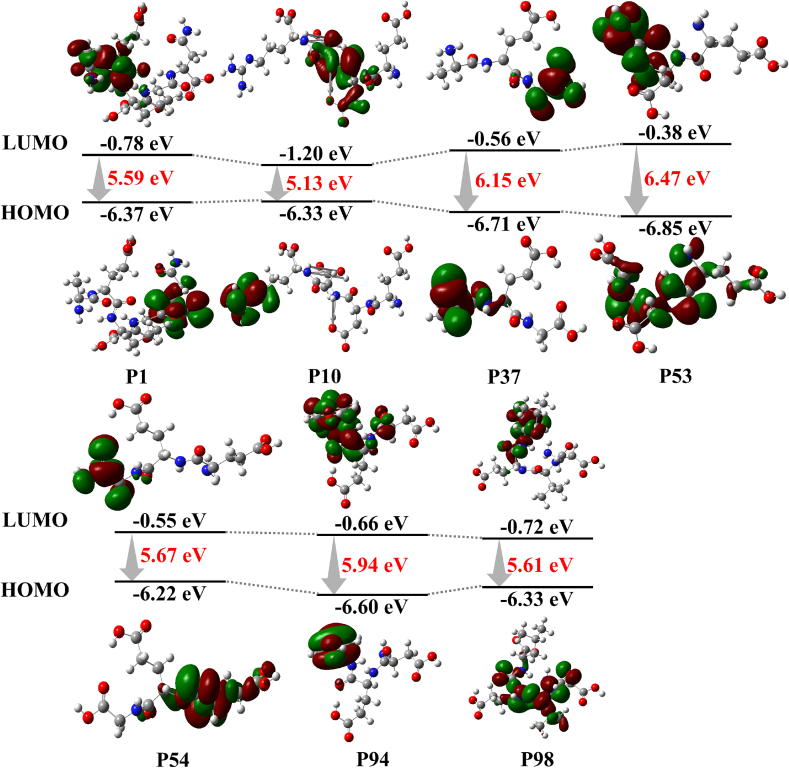
Isosurface visualization of frontier molecular orbitals for identified SCBC-derived novel antioxidant peptides compared with other candidates. The isosurfaces are illustrated in green and red lobes, which represent potential electron donor and accepter regions, respectively. HOMO, highest occupied molecular orbital; LUMO, lowest unoccupied molecular orbital. (For interpretation of the references to color in this figure legend, the reader is referred to the web version of this article.)

To elucidate the antioxidation-associated molecular structure attributes of P1, P10, and P98, quantum chemical computation was carried out using a DFT algorithm with other candidates for comparison. The profiles of frontier molecular orbitals are visualized in Fig. 5, and the calculated quantum properties for all peptides are shown in Table 2. The HOMO orbital is usually rich in electrons, allowing it to act as an antioxidative site by transferring electrons and quenching free radicals (Igbokwe et al., 2024). Meanwhile, a lower Δ*E* (HOMO-LUMO energy gap) generally implies easier electron transfer between HOMO and LUMO orbitals, demonstrating higher reactivity and preferable amphiphilic behavior of molecules (Mubango et al., 2025). P1, P10, and P98 had the smallest Δ*E* among all peptides, indicating a higher reactivity and electron donation potential than other candidates (Table 2 and Fig. 5), which was consistent with their strongest antioxidant activity among all candidates (Fig. 2, Fig. 3).

**Table 2:** Frontier orbital energies, active site distributions, and DFT global reactivity descriptors of identified SCBC-derived novel antioxidant peptides compared with other candidates.

Parameter		P1	P10	P37	P53	P54	P94	P98
Sequence		AEDVN	EDDR	AEG	EDG	EEG	DEY	DVEL
Frontier orbital energies (eV)	*E*_*HOMO*_	−6.37	−6.33	−6.71	−6.85	−6.22	−6.60	−6.33
	*E*_*LUMO*_	−0.78	−1.20	−0.56	−0.38	−0.55	−0.66	−0.72
	Δ*E*	5.59	5.13	6.15	6.47	5.67	5.94	5.61
Active site distribution	Residue	N5	R4	A1	E1	E1	Y3	D1
	Key group	Acylamino	Guanidinium	Amino	Amino	Amino	Hydroxyphenyl	Amino
	Active site	N_38_-H_40_	N_32_-H_71_	N_5_-H_26_	N_9_-H_30_	N_9_-H_30_	O_24_-H_2_	N_8_-H_9_
	Bond length (Å)	1.01	1.02	1.02	1.02	1.02	0.97	1.02
	Charge difference	1.08	1.07	1.01	1.06	1.05	1.05	1.10
	Key atom	N_38_	N_32_	N_5_	N_9_	N_9_	O_24_	N_8_
DFT global reactivity descriptors (eV)	*I*	6.37	6.33	6.71	6.85	6.22	6.60	6.33
	*A*	0.78	1.20	0.56	0.38	0.55	0.66	0.72
	*μ*	−3.58	−3.77	−3.64	−3.62	−3.39	−3.63	−3.53
	*χ*	3.58	3.77	3.64	3.62	3.39	3.63	3.53
	*η*	2.80	2.57	3.08	3.24	2.84	2.97	2.81
	*Ѕ*	0.18	0.19	0.16	0.15	0.18	0.17	0.18
	*ω*	2.29	2.76	2.15	2.02	2.02	2.22	2.21

*E*_*HOMO*_, HOMO orbital energy; *E*_*LUMO*_, LUMO orbital energy; Δ*E*, energy gap between HOMO and LUMO orbitals; *I*, ionization energy; *A*, electron affinity; *μ*, chemical potential, *χ*, electronegativity; *η*, chemical hardness; *S*, chemical softness; *ω*, electrophilicity; SCBC, silver carp bone collagen; DFT, density functional theory; HOMO, highest occupied molecular orbital; LUMO, lowest unoccupied molecular orbital.

According to the HOMO-LUMO orbital distributions, the redox active sites of peptide candidates were further analyzed, including key residues, groups, bonds, charges, and atoms. The primary active sites of P1 and P10 were predicted as the N_38_-H_40_ of acylamino on N5 and N_32_-H_71_ of guanidinium group on R4, respectively (Table 2). Consistently, Mubango et al. (2025) found that the key active sites of antioxidant peptides identified from fish maw hydrolysates, containing GFPGER, LLSGFRHR, FLLFRQ, ARSLNFEF, ERGFPGLP, and APDPFRHF, were all from guanidinium groups of R residues. The all sp^2^ hybridized C, N, and/or O allowed these chemical groups to possess p-orbital lone pairs at amino/imino N atoms, which were ready to donate a single electron for free radical quenching and simultaneously facilitate the groups to transform into a stable p-π conjugate structure (O=C(N•H)-, NH=C(N•H)-NH-, or NH=C(NH_2_)-N•-) (Igbokwe et al., 2024). Besides, the delocalized conjugate electrons also strongly polarized the amino/imino N–H bonds, benefiting the release of H^+^ or formation of stronger hydrogen bonds (Mubango et al., 2025), which further contributed to the good antioxidant activity of P1 and P10 (Fig. 2, Fig. 3). In addition, the main active sites of P98, P37, P53, and P54 were all predicted as the N–H of amino groups on N-terminal residues. Similarly, Liu, Liang, et al. (2022) also reported that the dominant active site of antioxidant peptide DDDY is N_10_-H_12_ of the amino group on the N-terminal D residue.

Moreover, seven conceptual DFT descriptive parameters were calculated based on the HOMO-LUMO energy profiles. Among these descriptors, *μ* represents the electronic flow from high potential (HOMO) to low potential (LUMO) (Igbokwe et al., 2024). Specifically, a lower *μ* value denotes a relatively higher reactivity and easier electronic transfer tendency across HOMO-LUMO orbitals (Mubango et al., 2025). Meanwhile, *η* is the resistance to lose electrons and is closely related to descriptors *I* and *A* (Igbokwe et al., 2024). Molecules with a high *η* value are generally considered stable and less reactive with well-defined electron densities (Mubango et al., 2025). Hence, a low *μ* and *η* combined with a high *S* is commonly the characteristic of highly reactive molecules (Igbokwe et al., 2024; Mubango et al., 2025). P10 had the overall smallest *μ*, lowest *η*, and highest *S* among all peptides, followed by P1 and P98 (lowest *η*, highest *S*, but moderate *μ*) (Table 2). This result was accordant with their lowest Δ*E* level (Table 2 and Fig. 5) and strongest antioxidation (Fig. 2, Fig. 3) among all candidates, and suggested that the good electron donation characteristic can be a crucial contributor to their strong antioxidation.

### 3.8 Underlying interactive mechanism between identified peptides and Keap1

**Fig. 6:**
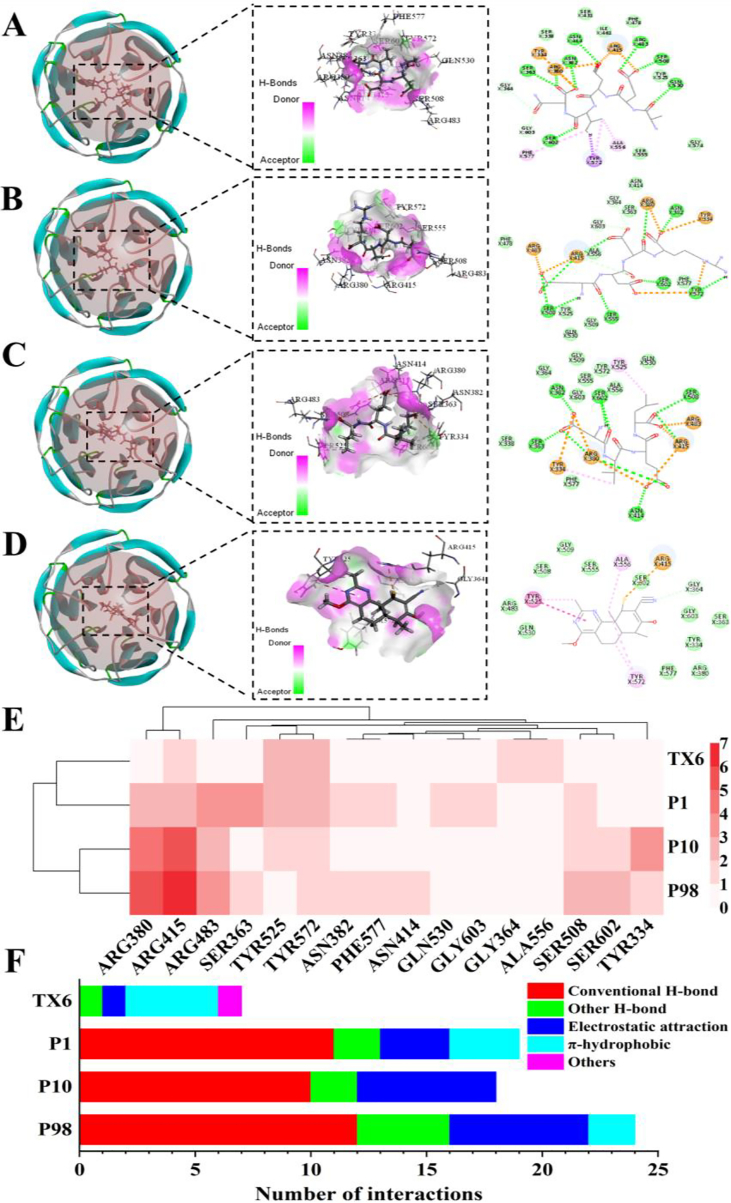
Underlying interactive mechanism of identified SCBC-derived novel antioxidant peptides with Keap1 revealed by molecular docking. (A–D) 3D and 2D diagrams of P1 (A), P10 (B), P98 (C), and TX6 (D, reference ligand) binding with the active center of Keap1, respectively. Non-bonding interaction mode: , conventional hydrogen bond; , carbon hydrogen bond/π-donor hydrogen bond; , attractive charge/salt bridge/π-anion; , π-σ; , alkyl/π-alkyl. E, HCA analysis of non-bonding interactive sites between the above ligands and Keap1. The color gradation represented the number of non-bonding interactions related with the given amino acid residues. F, statistics of non-bonding interaction modes for each above ligand interacted with Keap1. SCBC, silver carp bone collagen.

The underlying mechanism of the identified novel antioxidant peptides with Keap1 can be elucidated at the molecular level through molecular docking. Fig. 6A-C shows the non-bonding interactions of identified peptides binding to Keap1. The active center of Keap1 is a shallow pocket of the Kelch domain, allowing the ETGE motif of Nfr2 to insert in a folding pattern of β-turn, which forms a stable Keap1-Nfr2 complex (Mubango et al., 2025). Meanwhile, Huerta et al. (2016) reported that TX6 could bind with Keap1, activate Nrf2, and thereby promote ARE expression. Hence, TX6 was used as a reference ligand for comparative analysis of Keap1-binding patterns, and its non-bonding interactive diagram is displayed in Fig. 6D. It has been shown in Table 1 that the –CIE values of P1, P10, and P98 docking with Keap1, which represented their binding affinity, were 107.51, 107.71, and 103.19 kcal/mol, respectively, much higher than that of TX6 (29.67 kcal/mol) and other candidates.

Moreover, the non-bonding interactive sites and modes were further statistically analyzed with results illustrated in Fig. 6E and Fig. 6F, respectively. TX6 predominantly bound with Keap1 through hydrophobic interactions, including a π-π t-shaped interaction, three π-alkyl interactions, and an alkyl interaction (Fig. 6F), which were mainly contributed by the heterocycle and cycloalkane structure of TX6 (Fig. 6D). However, the identified peptides mainly bound with Keap1 by hydrogen bonds and electrostatic attractions, containing 10–12 conventional hydrogen bonds, 2–4 carbon hydrogen bonds, 3–6 attractive charges/salt bridges, which were primarily attributed to numerous amide, carboxyl, and amino groups of these peptides (Fig. 6A-C). Besides, ARG415, TYR525, TYR572, GLY364, and ALA556 were the interactive sites for TX6 to bind with Keap1 (Fig. 6E). Nevertheless, the core interactive sites of identified peptides were extremely different, mainly involving ARG380, ARG415, ARG483, TYR572, and SER508 (Fig. 6E). Interestingly, many previous studies also reported similar binding modes and sites with Keap1 for antioxidant peptides identified from various sources, such as snakehead soup (Zhang, Li, et al., 2021), fish maw (Mubango et al., 2025), pork sausage (Zou et al., 2023), and yak milk protein (Wang, Danzeng, et al., 2025). Overall, the numerous amide, carboxyl, and amino groups allowed identified peptides to form strong hydrogen bonds and electrostatic attractions with core residues of the Kelch domain within Keap1 (such as ARG380, ARG415, and ARG483), thereby conducting antioxidation through activating the Keap1-Nfr2-ARE pathway.

### 3.9 Potential antioxidative targets and PPI network of identified peptides

As the benefits of antioxidants on human health could be attributed to their widely ranged bioactivities associated with antioxidation (Wang et al., 2024), the antioxidant-related targets and PPI network of P1, P10, and P98 were predicted through network pharmacology analysis, with results presented as Fig. S2A and Fig. S2B, respectively. 100 putative targets were found for each identified peptide, while 1330 antioxidation-associated targets with a relevance score > 1.0 were obtained. Afterward, a total of 49 intersected target proteins for all identified peptides were found through alignment, among which 19 targets jointly belonged to P1, P10, and P98. These intersected targets could form a PPI network comprising 48 nodes (without disconnected nodes) and 226 edges (Fig. S2B). Subsequently, these intersected targets were further ranked based on 8 topological analysis algorithms, including MCC, MNC, EPC, Degree, Closeness, Betweenness, Radiality, and Stress, with results shown in Fig. S2C–D. Then the top 10 significant targets from each algorithm were filtered, among which six targets, namely PTGS2 (prostaglandin-endoperoxide synthase 2), SIRT1 (sirtuin 1), MMP2 (matrix metallopeptidase 2), CASP3 (caspase 3), PPARG (peroxisome proliferator activated receptor γ), and MMP9, were found overlapped for all algorithms and considered the hub targets of identified antioxidant peptides (Fig. S2D).

Furthermore, among these hub targets, MMP9, CASP3, and PPARG had the most connections with other antioxidative target proteins (Fig. S2B and Fig. S2D). It is reported that the MMP9 up-regulation induced by ROS could accelerate extracellular matrix degradation and stimulate inflammatory response, which promotes further tissue damage and free radicals' generation (Lin et al., 2021). Additionally, PPARG, activated by fatty acid derivatives, could not only facilitate Nrf2 transcription to up-regulate the Keap1-Nrf2-ARE pathway but also inhibit the NF-κB (nuclear factor κ-light-chain-enhancer of activated B cells) pathway to down-regulate MMP9 expression, thereby alleviating oxidative stress (Wang et al., 2020). Besides, CASP3 can induce apoptosis *via* the mitochondrial pathway to renew cells subjected to severe oxidative damage, and could also be inhibited by the Keap1-Nrf2-ARE pathway through up-regulating downstream heme oxygenase-1, protecting cells from mild oxidative stress-induced apoptosis (Lin et al., 2021). Wang et al. (2020) reported that antioxidant peptide KHNRGDEF from rice bran up-regulated the expression of PPARG and Nrf2 to relieve oxidative stress in aged mice. Lin et al. (2021) found that crude chalaza hydrolysates could perform antioxidation in damaged hepatocytes by decreasing MMP2 and MMP9 activities as well as increasing the cleaved CASP3 level. These reports were both accordant with our above findings.

### 3.10 Protective pathways against oxidative stress based on GO and KEGG analyses

To further explore the protective pathways of P1, P10, and P98 against oxidative stress, GO annotation and KEGG enrichment analyses were carried out using the predicted key antioxidant targets. The top 10 entries with the most targets and *P* < 0.01 in BP, CC, and MF categories of GO analysis are shown in Fig. S2E. In the BP category, these targets mainly focused on proteolysis (40.82%), positive regulation of apoptotic process (26.53%), and apoptotic process (22.45%). In terms of CC, they were mostly distributed in cytosol (61.22%), cytoplasm (57.14%), and plasma membrane (46.94%). For the MF entry, they were dominantly associated with protein binding (87.76%) and metal ion binding (38.78%), corresponding to their high Keap1-binding affinity (Table S1) and Fe^2+^-chelating capacity (Fig. 2E) of identified peptides, which suggested their potency to exert cytoprotection against oxidative stress by clearing transition metal ions and regulating activities of antioxidant-related proteins.

Furthermore, the top 20 pathways with the highest enrichment factors and *P* < 0.01 are displayed in Fig. S2F. These targets were primarily enriched in pathways associated with cancer (28.57%), lipid and atherosclerosis (24.49%), and apoptosis (20.41%), such as NF-κB, TNF (tumor necrosis factor), and IL-17 (interleukin-17) signaling pathways. Meanwhile, five hub targets (excluding only SIRT1) were involved in these three pathways. Consistently, Wang et al. (2024) and Wang, Danzeng, et al. (2025) found that antioxidant peptides from soy protein and yak milk, respectively, showed the most significantly KEGG-enriched target pathways containing lipid and atherosclerosis as well as pathways in cancer. Atherosclerosis is considered closely related to the oxidative damage of vascular endothelial cells (Wang, Danzeng, et al., 2025), and apoptosis is often attributed to oxidative stress through various pathways (Lin et al., 2022). Overall, the identified novel antioxidant peptides potentially further target pathways with respect to cancer, lipid metabolism, and cardiovascular disease for redox modulation in the human body, which requires further experimental verification.

## 4 Conclusions

This work has achieved, for the first time, the unveiling of SCBC-derived novel antioxidant peptides and their underlying multi-dimensional mechanism through an integrated strategy of *in silico*, *in vitro*, and *in cellulo* approaches. Results showed that “chymotrypsin A + ficin” can be an optimal combined-enzymatic strategy through simulated proteolysis, allowing 179 peptides to be released from SCBC, among which seven peptides were selected as antioxidant candidates by virtual screening. AEDVN, EDDR, and DVEL were finally identified as novel antioxidant peptides through both *in vitro* assessment and *in cellulo* validation. They showed good DPPH radical-scavenging activity (IC_50_ = 0.89, 2.59, and 1.40 mM, respectively), strong Fe^2+^-chelating ability (IC_50_ = 0.98, 1.39, and 3.72 mM, respectively), high stability against SGID (82.99–94.29% activity remaining), and non-cytotoxicity (200–1000 μM) in HepG2 cells. Quantum chemical analysis demonstrated that the acylamino, guanidinium, and N-terminal amino groups were their key redox active sites, respectively. Molecular docking results indicated that they can bind with core residues of the Kelch domain within Keap1 (such as ARG380, ARG415, and ARG483) by strong hydrogen bonds and electrostatic attractions, thereby activating the Keap1-Nfr2-ARE antioxidant pathway. Hence, the activities of antioxidant enzymes SOD, CAT, and GSH-Px were significantly increased, resulting in the production decline of MDA and subsequent viability improvement of H_2_O_2_-induced oxidatively damaged cells. Furthermore, network pharmacological analysis suggested their underlying *in vivo* antioxidant pathways beyond the Keap1-Nrf2-ARE pathway (such as NF-κB, TNF, and IL-17 signaling pathways) by regulating multiple targets (such as MMP9, CASP3, and PPARG). This work provided theoretical support for the high-value utilization of freshwater fish by-products and help understand the multi-dimensional antioxidation mechanism of collagen peptides.

## CRediT authorship contribution statement

**Yimeng Mei:** Writing – original draft, Visualization, Methodology, Investigation. **Feng Lin:** Formal analysis. **Ruoyu Xie:** Validation, Methodology. **Jiaxin Chen:** Validation, Investigation. **Jun Hu:** Software. **Wenxuan Chen:** Funding acquisition. **Hongying Du:** Conceptualization. **Guijie Hao:** Resources. **Shuangxi Li:** Resources. **Jin Zhang:** Writing – review & editing, Writing – original draft, Visualization, Supervision, Methodology, Funding acquisition, Conceptualization.

## Declaration of competing interest

The authors declare that they have no known competing financial interests or personal relationships that could have appeared to influence the work reported in this paper.

## Data Availability

Data will be made available on request.
