# Supplementary material for: Unveiling novel antioxidant peptides from silver carp (Hypophthalmichthys molitrix) bone collagen and underlying multi-dimensional mechanism by integrating in silico, in vitro, and in cellulo approaches
Source: Food Chem X. 2026 Jun 6;37:104070. doi: 10.1016/j.fochx.2026.104070 (PMC13273142; doi:10.1016/j.fochx.2026.104070)
Supplement: Supplementary material [file mmc1.docx]

**Unveiling novel antioxidant peptides from silver carp (*Hypophthalmichthys molitrix*) bone collagen and underlying multi-dimensional mechanism by integrating *in silico*, *in vitro*, and *in cellulo* approaches**

Yimeng Mei ^a,^ †; Feng Lin ^c,^ †; Ruoyu Xie ^a^; Jiaxin Chen ^a^; Jun Hu ^a^; Wenxuan Chen ^a^; Hongying Du ^b^; Guijie Hao ^c^; Shuangxi Li ^d,^ *; Jin Zhang ^a,^ *

^a^ State Key Laboratory for Quality and Safety of Agro-products, Zhejiang Key Laboratory of Intelligent Food Logistic and Processing, Institute of Food Science, Zhejiang Academy of Agricultural Sciences, Hangzhou, Zhejiang 310021, P. R. China;

^b^ Department of Food Science and Engineering, College of Light Industry and Food Engineering, Nanjing Forestry University, Nanjing 210037, P. R. China;

^c^ Key Laboratory of Healthy Freshwater Aquaculture, Ministry of Agriculture and Rural Affairs, Key Laboratory of Fish Health and Nutrition of Zhejiang Province, Huzhou Key Laboratory of Aquatic Product Quality Improvement and Processing Technology, Zhejiang Institute of Freshwater Fisheries, Huzhou, Zhejiang 313001, P. R. China;

^d^ Xingzhi College, Zhejiang Normal University, Lanxi, Zhejiang 321100, P. R. China

† These authors contributed equally to this study as co-first authors.

* Corresponding authors: Jin Zhang, Research Associate Professor; Shuangxi Li, Associate Professor.

1. Institute of Food Science/State Key Laboratory for Quality and Safety of Agro-products, Zhejiang Academy of Agricultural Sciences, No.198 Shiqiao Road, Shangcheng District, Hangzhou, Zhejiang 310021, P. R. China. Tel & Fax: +86-571-85131729. E-mail: [zhangjin@zaas.ac.cn](mailto:zhangjin@zaas.ac.cn) (J. Zhang).

2. Xingzhi College, Zhejiang Normal University, No.3388 Yingbin Road, Lanxi city, Jinhua, Zhejiang 321100, P. R. China. Tel & Fax: +86-579-82291190. E-mail: [lsx@zjnu.cn](mailto:lsx@zjnu.cn) (S. Li).


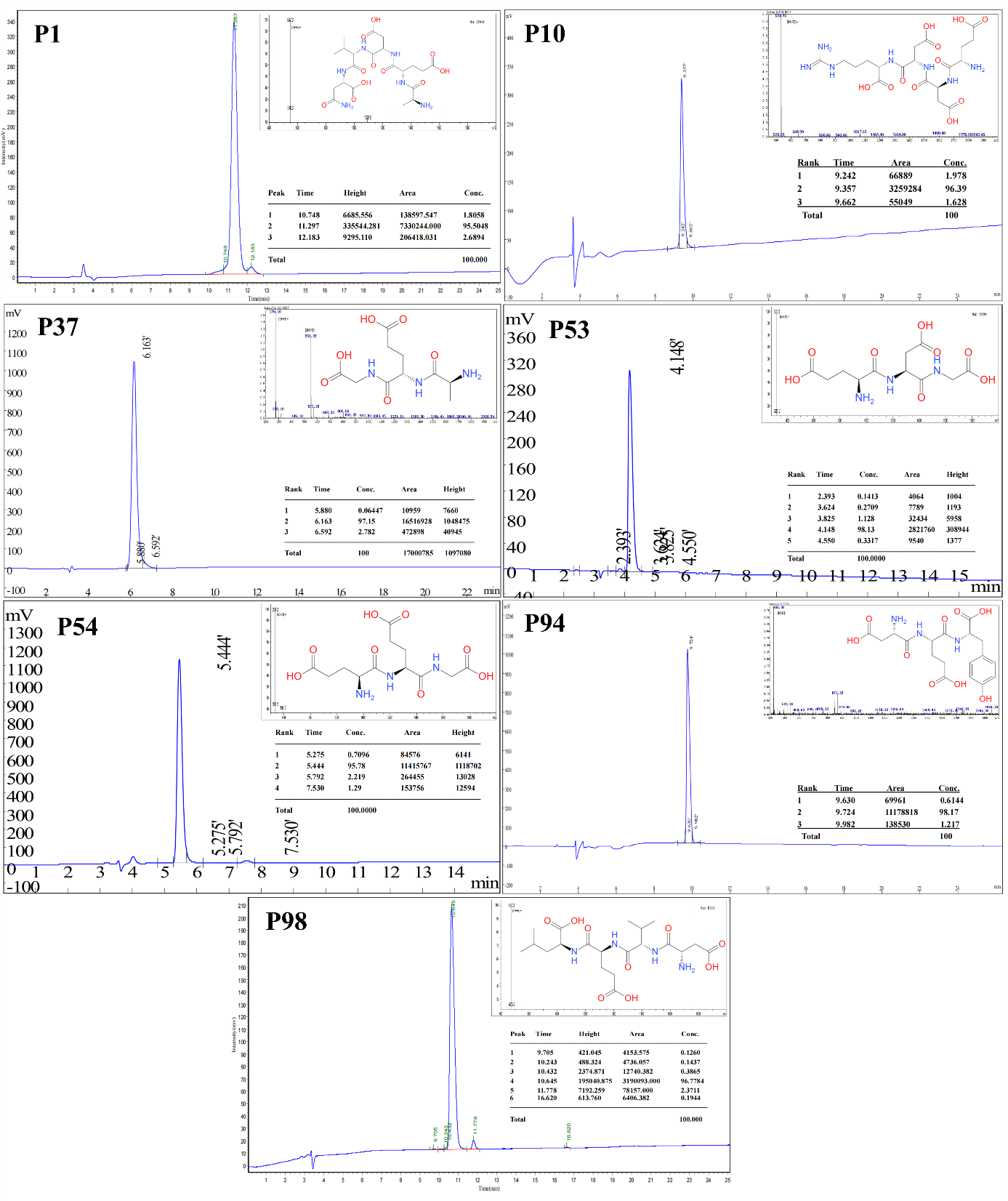


**Fig. S1. HPLC chromatograms and MS spectra of synthesized novel antioxidant peptide candidates derived from SCBC.** Each master figure showed the HPLC chromatogram and purity of synthesized peptide, while the attached figure showed the MS spectrum and structural formula of synthesized peptide.
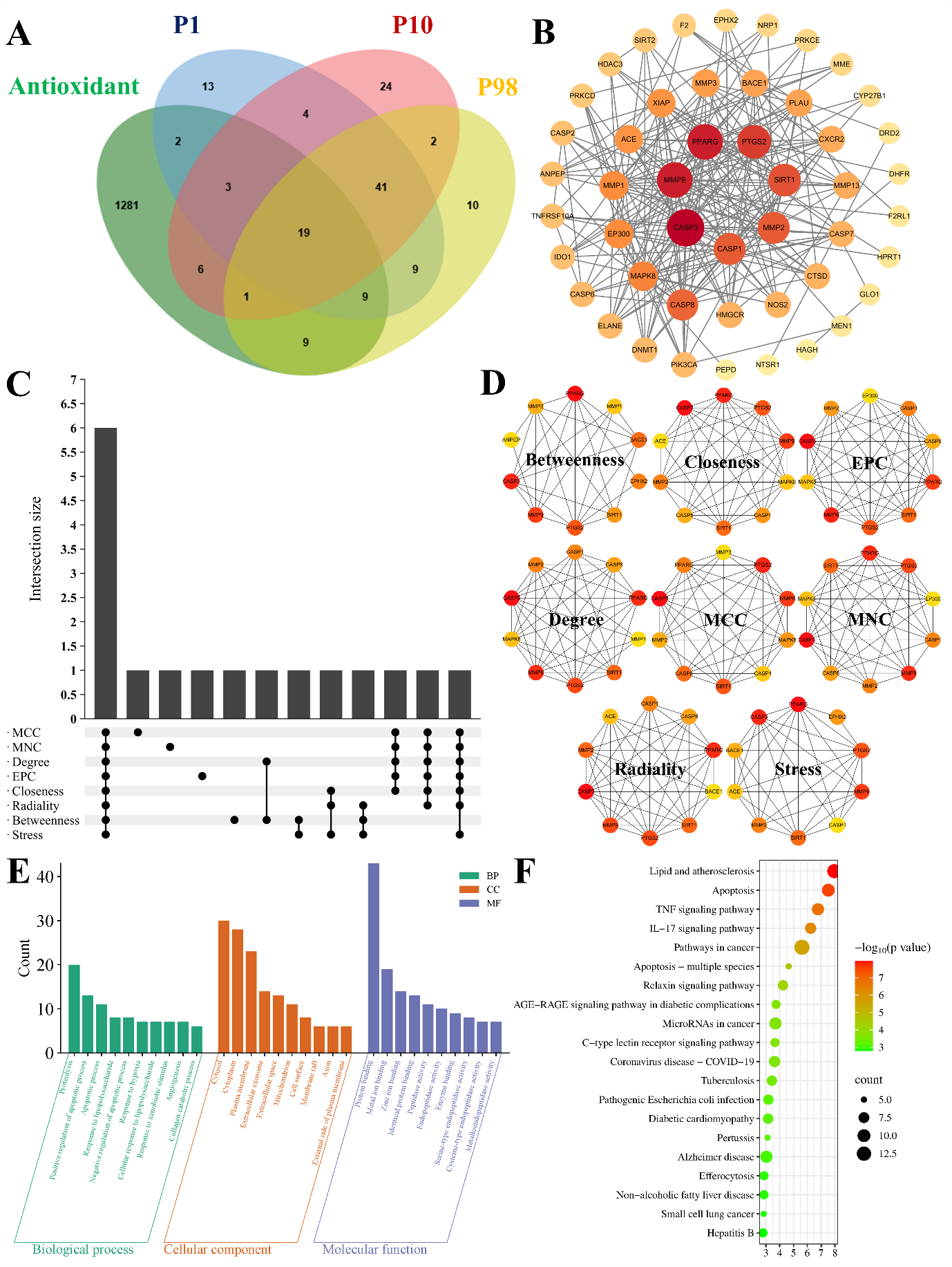


**Fig. S2. Antioxidative targets, protein-protein interaction (PPI) network, Gene Ontology (GO) annotation, and Kyoto Encyclopedia of Genes and Genomes (KEGG) pathway enrichment analyses of identified SCBC-derived novel antioxidant peptides.** A, Venn diagram of intersected targets between identified peptides and antioxidation. B, PPI network map of antioxidative targets for identified peptides; C-D, intersection plot (C) and network map (D) of top 10 key antioxidative targets obtained through 8 topological analysis algorithms; E, GO functions of antioxidative targets; F, KEGG enriched pathways of antioxidative targets. In (B) and (D), the red color indicates more edges while the yellow color represents fewer edges. BP, biological process; CC, cellular component; MF, molecular function.

**Table S1 Virtual screening of SCBC-derived novel antioxidant peptide candidates based on their potential of** **binding with Keap1, scavenging free radicals, and chelating transition metal ions**

| Peptide | Sequence | –CIE (kcal/mol) | Score A | FRS | Score B | CHEL | Score C | Total score |
| --- | --- | --- | --- | --- | --- | --- | --- | --- |
| **P1** | **AEDVN** | **107.51** | **99.72** | **0.31** | **80.00** | **0.23** | **81.81** | **90.31** |
| P2 | AM | 43.69 | 63.63 | 0.40 | 88.55 | 0.27 | 90.25 | 76.52 |
| P3 | CN | 45.72 | 64.78 | 0.41 | 89.89 | 0.27 | 91.09 | 77.64 |
| P4 | CTL | 60.25 | 73.00 | 0.38 | 86.50 | 0.25 | 86.59 | 79.77 |
| P5 | CTS | 56.46 | 70.85 | 0.35 | 83.96 | 0.25 | 86.34 | 78.00 |
| P6 | DL | 65.31 | 75.86 | 0.37 | 85.97 | 0.28 | 91.96 | 82.41 |
| P7 | DN | 63.67 | 74.93 | 0.37 | 86.30 | 0.28 | 91.82 | 81.99 |
| P8 | DTG | 77.22 | 82.59 | 0.38 | 87.24 | 0.26 | 87.69 | 85.03 |
| P9 | DVW | 78.03 | 83.05 | 0.41 | 89.47 | 0.22 | 81.14 | 84.18 |
| **P10** | **EDDR** | **107.71** | **99.83** | **0.35** | **83.91** | **0.24** | **84.44** | **92.00** |
| P11 | EVG | 78.39 | 83.25 | 0.40 | 88.78 | 0.24 | 85.03 | 85.08 |
| P12 | EY | 71.35 | 79.28 | 0.51 | 99.30 | 0.25 | 85.86 | 85.93 |
| P13 | IK | 55.15 | 70.12 | 0.36 | 84.54 | 0.25 | 85.94 | 77.68 |
| P14 | IM | 52.30 | 68.50 | 0.39 | 88.20 | 0.27 | 91.07 | 79.07 |
| P15 | IQG | 58.72 | 72.14 | 0.41 | 89.71 | 0.27 | 89.19 | 80.79 |
| P16 | IQL | 70.79 | 78.96 | 0.37 | 85.49 | 0.26 | 88.94 | 83.09 |
| P17 | PDW | 79.31 | 83.77 | 0.48 | 96.11 | 0.26 | 88.40 | 88.02 |
| P18 | PIIDIAPM | 80.42 | 84.40 | 0.33 | 82.13 | 0.22 | 81.47 | 83.10 |
| P19 | PQM | 63.95 | 75.09 | 0.44 | 92.19 | 0.28 | 92.88 | 83.81 |
| P20 | PTES | 79.24 | 83.74 | 0.35 | 84.16 | 0.26 | 88.84 | 85.12 |
| P21 | QEF | 80.89 | 84.67 | 0.40 | 88.32 | 0.24 | 85.03 | 85.67 |
| P22 | QF | 56.08 | 70.64 | 0.40 | 89.17 | 0.27 | 90.51 | 80.24 |
| P23 | QK | 55.95 | 70.57 | 0.37 | 85.68 | 0.24 | 85.05 | 77.97 |
| P24 | QN | 56.50 | 70.88 | 0.39 | 88.13 | 0.27 | 90.98 | 80.22 |
| P25 | QVY | 63.90 | 75.06 | 0.44 | 92.81 | 0.22 | 80.49 | 80.86 |
| P26 | TDG | 71.68 | 79.46 | 0.39 | 88.07 | 0.26 | 87.45 | 83.61 |
| P27 | TEAS | 85.39 | 87.21 | 0.33 | 81.69 | 0.25 | 85.74 | 85.46 |
| P28 | TK | 49.49 | 66.92 | 0.36 | 85.23 | 0.25 | 85.83 | 76.22 |
| P29 | TS | 48.21 | 66.19 | 0.35 | 84.43 | 0.28 | 91.21 | 77.01 |
| P30 | TTK | 47.63 | 65.87 | 0.34 | 82.93 | 0.25 | 85.57 | 75.06 |
| P31 | VEG | 68.76 | 77.81 | 0.41 | 89.68 | 0.24 | 85.32 | 82.66 |
| P32 | VM | 50.51 | 67.49 | 0.39 | 88.20 | 0.26 | 88.68 | 77.97 |
| P33 | VQG | 63.37 | 74.76 | 0.41 | 89.66 | 0.25 | 86.81 | 81.50 |
| P34 | VTEDG | 100.67 | 95.85 | 0.36 | 85.08 | 0.22 | 80.00 | 89.20 |
| P35 | VVG | 60.61 | 73.20 | 0.38 | 86.89 | 0.23 | 82.99 | 79.07 |
| P36 | ADG | 66.48 | 76.52 | 0.39 | 87.66 | 0.25 | 86.75 | 81.86 |
| **P37** | **AEG** | **108.01** | **100.00** | **0.41** | **89.89** | **0.25** | **86.76** | **94.16** |
| P38 | AG | 45.57 | 64.70 | 0.42 | 90.70 | 0.27 | 90.34 | 77.61 |
| P39 | AK | 37.29 | 60.02 | 0.36 | 84.87 | 0.24 | 85.18 | 72.52 |
| P40 | APG | 51.13 | 67.84 | 0.46 | 94.89 | 0.29 | 93.57 | 81.03 |
| P41 | AQG | 57.78 | 71.60 | 0.41 | 89.89 | 0.26 | 88.38 | 80.37 |
| P42 | AR | 38.59 | 60.75 | 0.38 | 86.89 | 0.24 | 85.33 | 73.43 |
| P43 | AS | 45.69 | 64.76 | 0.35 | 84.01 | 0.27 | 90.47 | 76.00 |
| P44 | ATG | 31.75 | 56.88 | 0.39 | 88.23 | 0.25 | 87.08 | 72.27 |
| P45 | AVG | 55.13 | 70.10 | 0.38 | 87.10 | 0.24 | 84.37 | 77.92 |
| P46 | CK | 44.73 | 64.23 | 0.39 | 87.34 | 0.24 | 85.00 | 75.20 |
| P47 | DG | 48.75 | 66.50 | 0.41 | 89.52 | 0.27 | 90.88 | 78.35 |
| P48 | DIG | 74.05 | 80.80 | 0.37 | 85.59 | 0.25 | 86.41 | 83.40 |
| P49 | DK | 59.39 | 72.51 | 0.35 | 83.83 | 0.25 | 85.81 | 78.67 |
| P50 | DQG | 76.20 | 82.01 | 0.40 | 88.89 | 0.26 | 89.01 | 85.48 |
| P51 | DR | 54.27 | 69.62 | 0.37 | 85.86 | 0.25 | 85.94 | 77.76 |
| P52 | DS | 60.52 | 73.15 | 0.34 | 82.95 | 0.28 | 91.13 | 80.10 |
| **P53** | **EDG** | **94.31** | **92.26** | **0.41** | **89.37** | **0.26** | **87.52** | **90.35** |
| **P54** | **EEG** | **92.30** | **91.12** | **0.43** | **91.61** | **0.26** | **87.48** | **90.33** |
| P55 | EG | 61.36 | 73.63 | 0.44 | 92.31 | 0.27 | 91.07 | 82.66 |
| P56 | EIG | 81.18 | 84.83 | 0.39 | 88.23 | 0.25 | 86.40 | 86.07 |
| P57 | EK | 62.00 | 73.99 | 0.37 | 86.33 | 0.25 | 85.87 | 80.04 |
| P58 | EN | 64.13 | 75.19 | 0.40 | 88.98 | 0.28 | 91.93 | 82.82 |
| P59 | EPG | 75.86 | 81.83 | 0.48 | 96.73 | 0.29 | 94.31 | 88.67 |
| P60 | EQG | 80.50 | 84.45 | 0.43 | 91.60 | 0.26 | 89.17 | 87.42 |
| P61 | ER | 61.55 | 73.73 | 0.40 | 88.52 | 0.25 | 86.07 | 80.51 |
| P62 | ES | 64.81 | 75.58 | 0.37 | 85.63 | 0.28 | 91.36 | 82.03 |
| P63 | ETG | 79.76 | 84.03 | 0.41 | 89.87 | 0.26 | 87.77 | 86.42 |
| P64 | IAG | 57.94 | 71.69 | 0.40 | 88.46 | 0.27 | 89.30 | 80.29 |
| P65 | ITY | 64.77 | 75.55 | 0.44 | 92.68 | 0.24 | 84.00 | 81.95 |
| P66 | IVG | 60.28 | 73.02 | 0.38 | 86.85 | 0.24 | 85.25 | 79.53 |
| P67 | PAR | 60.91 | 73.37 | 0.42 | 90.37 | 0.28 | 91.18 | 82.07 |
| P68 | PDG | 70.68 | 78.90 | 0.44 | 92.59 | 0.28 | 92.64 | 85.76 |
| P69 | PG | 37.06 | 59.89 | 0.47 | 95.71 | 0.30 | 96.49 | 77.99 |
| P70 | PIG | 59.43 | 72.53 | 0.43 | 91.43 | 0.28 | 91.53 | 82.01 |
| P71 | PPG | 43.43 | 63.49 | 0.52 | 100.00 | 0.32 | 100.00 | 81.74 |
| P72 | PQG | 60.12 | 72.92 | 0.47 | 95.00 | 0.29 | 94.44 | 83.82 |
| P73 | PR | 39.58 | 61.31 | 0.43 | 91.79 | 0.27 | 91.05 | 76.36 |
| P74 | PS | 48.31 | 66.25 | 0.40 | 88.81 | 0.30 | 96.68 | 79.50 |
| P75 | PTG | 58.93 | 72.25 | 0.45 | 93.12 | 0.28 | 92.95 | 82.64 |
| P76 | PVG | 57.58 | 71.49 | 0.43 | 92.05 | 0.27 | 90.06 | 81.27 |
| P77 | QDG | 74.33 | 80.96 | 0.40 | 88.55 | 0.25 | 86.74 | 84.30 |
| P78 | QG | 51.92 | 68.29 | 0.43 | 91.51 | 0.27 | 90.10 | 79.55 |
| P79 | QR | 45.42 | 64.62 | 0.39 | 87.70 | 0.24 | 85.24 | 75.54 |
| P80 | TCR | 51.02 | 67.78 | 0.40 | 88.63 | 0.24 | 83.94 | 77.03 |
| P81 | TF | 49.72 | 67.04 | 0.40 | 88.71 | 0.28 | 91.36 | 78.54 |
| P82 | TG | 48.28 | 66.23 | 0.42 | 91.11 | 0.27 | 91.00 | 78.64 |
| P83 | TPG | 77.20 | 82.58 | 0.47 | 95.40 | 0.29 | 94.37 | 88.73 |
| P84 | VG | 49.74 | 67.05 | 0.42 | 90.30 | 0.26 | 88.77 | 78.29 |
| P85 | AF | 68.71 | 77.78 | 0.40 | 88.38 | 0.27 | 90.64 | 83.65 |
| P86 | AIG | 53.15 | 68.98 | 0.38 | 86.54 | 0.25 | 85.78 | 77.57 |
| P87 | AN | 46.05 | 64.97 | 0.38 | 87.29 | 0.28 | 91.18 | 77.10 |
| P88 | ATQL | 77.59 | 82.80 | 0.34 | 83.47 | 0.25 | 87.01 | 84.02 |
| P89 | AVL | 59.77 | 72.73 | 0.34 | 83.09 | 0.24 | 84.19 | 78.18 |
| P90 | AY | 46.77 | 65.38 | 0.49 | 97.63 | 0.24 | 85.12 | 78.38 |
| P91 | CDF | 73.25 | 80.35 | 0.39 | 87.84 | 0.24 | 85.10 | 83.41 |
| P92 | CS | 46.62 | 65.29 | 0.38 | 86.53 | 0.27 | 90.28 | 76.85 |
| P93 | CTM | 59.33 | 72.48 | 0.39 | 88.08 | 0.25 | 85.58 | 79.66 |
| **P94** | **DEY** | **95.18** | **92.75** | **0.45** | **93.71** | **0.24** | **83.66** | **90.71** |
| P95 | DIAPL | 90.76 | 90.25 | 0.39 | 87.34 | 0.27 | 90.41 | 89.56 |
| P96 | DPG | 71.01 | 79.08 | 0.45 | 93.92 | 0.29 | 94.22 | 86.58 |
| P97 | DTS | 77.33 | 82.66 | 0.32 | 80.68 | 0.25 | 87.08 | 83.27 |
| **P98** | **DVEL** | **103.19** | **97.27** | **0.35** | **83.56** | **0.25** | **85.77** | **90.97** |
| P99 | DY | 60.59 | 73.19 | 0.48 | 96.52 | 0.25 | 85.67 | 82.15 |
| P100 | ETIN | 91.91 | 90.90 | 0.34 | 83.45 | 0.26 | 88.63 | 88.47 |
| P101 | IG | 48.88 | 66.57 | 0.42 | 90.42 | 0.28 | 91.20 | 78.69 |
| P102 | IW | 54.85 | 69.95 | 0.48 | 96.11 | 0.26 | 87.35 | 80.84 |
| P103 | PEG | 71.02 | 79.09 | 0.46 | 94.85 | 0.28 | 92.61 | 86.41 |
| P104 | PEW | 60.02 | 72.87 | 0.50 | 98.43 | 0.26 | 88.39 | 83.14 |
| P105 | PQS | 52.80 | 68.79 | 0.39 | 87.85 | 0.29 | 93.83 | 79.81 |
| P106 | PL | 47.42 | 65.74 | 0.43 | 91.90 | 0.31 | 97.55 | 80.23 |
| P107 | PN | 49.13 | 66.71 | 0.44 | 92.21 | 0.31 | 97.39 | 80.76 |
| P108 | QL | 43.39 | 63.47 | 0.39 | 87.88 | 0.28 | 91.21 | 76.50 |
| P109 | QAG | 64.44 | 75.37 | 0.41 | 89.65 | 0.26 | 88.28 | 82.17 |
| P110 | QEK | 69.42 | 78.18 | 0.36 | 84.95 | 0.24 | 84.43 | 81.44 |
| P111 | QS | 48.24 | 66.21 | 0.36 | 84.81 | 0.27 | 90.38 | 76.90 |
| P112 | QTG | 66.15 | 76.34 | 0.40 | 89.10 | 0.25 | 86.95 | 82.18 |
| P113 | QW | 56.28 | 70.76 | 0.49 | 97.29 | 0.25 | 86.16 | 81.24 |
| P114 | TEF | 75.28 | 81.50 | 0.39 | 87.96 | 0.25 | 85.84 | 84.20 |
| P115 | TN | 47.25 | 65.65 | 0.39 | 87.69 | 0.28 | 91.88 | 77.72 |
| P116 | TR | 43.30 | 63.42 | 0.38 | 87.15 | 0.25 | 85.91 | 74.97 |
| P117 | VAG | 56.56 | 70.91 | 0.40 | 88.47 | 0.25 | 86.89 | 79.30 |
| P118 | VK | 48.22 | 66.20 | 0.36 | 84.54 | 0.24 | 83.75 | 75.17 |
| P119 | VL | 33.15 | 57.68 | 0.38 | 86.74 | 0.27 | 89.68 | 72.94 |

–CIE, the –CDOCKER interaction energy by molecular docking with Keap1 through DS2019; FRS, the free radical scavenging potential by AnOxPePred V1.0; CHEL, the transition metal ion chelating potential by AnOxPePred V1.0.

**Table S2 Physicochemical properties of identified SCBC-derived novel antioxidant peptides compared with other candidates**

| **Property** | **P1** | **P10** | **P37** | **P53** | **P54** | **P94** | **P98** |
| --- | --- | --- | --- | --- | --- | --- | --- |
| Sequence | AEDVN | EDDR | AEG | EDG | EEG | DEY | DVEL |
| Source and location | α1(I) [1329-1333] | α1(I) [25-28] | α1(I) [348-350, 924-926, 1384-1386], α2(I) [1288-1290] | α1(I) [216-218], α2(I) [133-135, 1300-1302] | α1(I) [450-452], α2(I) [367-369] | α2(I) [1105-1107] | α2(I) [1283-1286] |
| MW (Da) | 546.30 | 533.15 | 275.10 | 319.20 | 333.20 | 425.05 | 474.30 |
| pI | 2.89 | 3.67 | 3.21 | 2.99 | 3.04 | 2.92 | 2.98 |
| Net charge | -2 | -2 | -1 | -2 | -2 | -2 | -2 |
| Hydrophobicity (kcal /mol) | 16.06 | 20.62 | 13.18 | 16.32 | 16.31 | 14.46 | 13.46 |
| Sensory quality | salty, umami | umami | umami | umami | umami | umami | bitter, sour, umami |
| Water solubility (mM) | 39.81 | 38.90 | 109.65 | 75.86 | 70.79 | 21.38 | 56.23 |

MW, molecular weight; pI, isoelectric point.
